# Supplementary material for: Critical evaluation of drug response prediction models with DrEval
Source: Nat Commun. 2026 May 12;17:4238. doi: 10.1038/s41467-026-72903-w (PMC13168506; doi:10.1038/s41467-026-72903-w)
Supplement: Supplementary file 1 — Supplementary Information [file 41467_2026_72903_MOESM1_ESM.pdf]

Critical Evaluation of Drug Response Prediction Models with DrEval

J. Bernett, P. Iversen, M. Picciani, M. Wilhelm, K. Baum, M. List

Supplementary Information

1 Supplementary Discussion

1.1 Cross-study prediction on GDSC1, GDSC2, BeatAML2, and PDX_Bruna

We assessed how model performance is affected when both the input features and the output domain (i.e., drug response measurements) differ from the training data distribution. This scenario applies to GDSC1, GDSC2, BeatAML2, and PDX_Bruna. For GDSC1 and GDSC2, gene expression and methylation data are primarily available as microarray and Illumina BeadChip measurements, respectively, rather than the RNA-seq and RRBS data used during training. Cross-study performance using RNA-seq and RRBS inputs on these datasets has been previously explored [1, 2]. However, substantial differences exist between the platforms: RMA-normalized microarray expression data is not only limited in feature coverage but also lies on a different scale compared to RNA-seq TPMs (Figure S7), and RRBS CpG clusters do not directly correspond to BeadChip-defined CpG islands. To address this, we mapped RRBS cluster regions to the closest matching BeadChip islands based on maximum overlap, discarding clusters without a suitable match or where a more compatible cluster existed (details in the data preprocessing GitHub repository `https://github.com/daisybio/preprocess_drp_data`).

Among all cell line datasets, GDSC1 exhibits the highest cross-study prediction errors, likely due to the additional challenge posed by its use of a different viability assay (Syto60), in contrast to CellTiter-Glo used in all other datasets. This makes generalization to GDSC1 particularly difficult, as it involves both out-of-distribution inputs and a shift in the output distribution. Overall, prediction errors on GDSC1 and GDSC2 are substantially higher than on CTRPv1 and CCLE (as expected), as none of the model weights are tuned to this domain.

Prediction errors on the ex vivo datasets are substantially higher than those observed for the cell-line datasets (Table S8), a pattern that holds consistently across all response measures (Table S9). As shown in Figure S9, the $lnIC50$ and $pEC50$ distributions for BeatAML2 and PDX_Bruna are shifted relative to CTRPv2 but remain broadly comparable to those of the other cell-line screens. In contrast, the AUC values for BeatAML2 are generally higher than in any other dataset, which likely contributes to the particularly poor cross-study performance for AUC predictions reported in Table S9. The gene expression inputs also exhibit distributional shifts: BeatAML2 (RNA-seq) differs from CTRPv2 (RNA-seq) but is more aligned with the microarray-based GDSC1 and GDSC2 datasets. BeatAML2 further contains several low-value outliers. Methodological differences may also play a role, as BeatAML2 was measured with CellTiter-96, whereas PDX_Bruna used CellTiter-Glo. Taken together, these factors help explain why performance on BeatAML2 is even worse than on PDX_Bruna. However, the magnitude of the distributional shifts is not substantially larger than the shifts observed between CTRPv2 and GDSC1/2, suggesting that assay and input differences alone do not account for the performance degradation on the ex vivo datasets. It is more likely that immortalized cell lines respond fundamentally differently from ex vivo patient/PDX-derived cells. This observation is critical, as one of the central aims of drug response modeling is to predict responses in patients. Although ex vivo assays are biologically closer to clinical samples than cell-line screens, they remain artificial experimental systems. In real-world clinical data, we, for example, cannot measure $IC50$s, $EC50$s, or AUCs. The models already perform poorly in this intermediate setting, indicating that naive transfer to clinical data is unlikely to work.

1.2 Simpson's Paradox in Drug Response Prediction

In the manuscript, we describe a widespread issue in the evaluation of drug response models, which we interpret as a form of Simpson's Paradox. Simpson's Paradox is a phenomenon in statistics where trends observed within individual groups can differ substantially from the trend observed in the combined data. Classical examples of it show cases where trends disappear or reverse during group combination [3]. In the case of drug response modeling, the trend is the correlation of the predicted with the ground truth response, which considerably strengthens when analyzed over all drugs, instead of for each drug separately.

Let y_{ij} denote the drug response of cell line i to drug j. We decompose the drug response into

$$y_{ij} = d_j + c_i + (dc)_{ij} + \epsilon_{ij},$$

where d_j is the drug effect, c_i is the cell line effect, $(dc)_{ij}$ is an interaction effect between drug and cell line, and ϵ_{ij} is noise. For personalized medicine applications, the drug effects can be trivially estimated from the training data as the mean response over all cell lines for each drug (NaiveDrugMeanPredictor, $\hat{y}_{ij} = d_j$). The explained variance of a naive drug mean predictor can then be expressed as

$$R^2_{\text{global}}(\text{NaiveDrugMeanPredictor}) = \frac{\sigma_d^2}{\sigma_{total}^2} = \frac{\sigma_d^2}{\sigma_d^2 + \sigma_c^2 + \sigma_{dc}^2 + \sigma_\epsilon^2},$$

where σ_d^2, σ_c^2, σ_{dc}^2, σ_ϵ^2, and σ_{total}^2 denote variances of drug effects, cell line effects, their interaction, residual noise, and total variance, respectively. However, in the drug response datasets we analyzed, the drugs vary strongly in their overall $IC50$, i.e., the drug effects dominate:

$$\sigma_d^2 \gg \sigma_c^2 + \sigma_{dc}^2 + \sigma_\epsilon^2,$$

which pushes the R^2 of a naive (and useless) model over 80 % for CTRPv2. Therefore, all models that manage to estimate the drug effects get a misleadingly high R^2 by default. It is crucial, instead, to estimate cell line and interaction effects for an unseen cell line. This is the actual interesting signal, answering: "How does this unseen cell line react to cancer drugs overall and how does it react to a given drug specifically?" We get a much more meaningful measure by looking at the explained variance of ground truth and prediction within each drug, because this removes the dominating but trivial drug effect. The variance of the drug response of one drug that needs to be explained is: $\text{Var}(y_{i|d_j}) = \sigma_c^2 + \sigma_{dc}^2 + \sigma_\epsilon^2$. This leaves only meaningful contributions that can not be trivially explained by remembering train set characteristics. Simpson's Paradox then manifests, because current models explain little of the within-drug variance $\text{Var}(y_{i|d_j})$. For the NaiveDrugMeanPredictor, this trivially leads to an R^2 of zero for each drug.

$$R^2_{\text{drug j}}(\text{NaiveDrugMeanPredictor}) = \frac{0}{\sigma_c^2 + \sigma_{dc}^2 + \sigma_\epsilon^2}.$$

We show a simple synthetic example of this effect using just two drugs and 5 cell lines in Figure S1.

1.3 Difficulties in Extending Predictions to New Drugs

We have shown that all evaluated models struggle to generalize to unseen drugs, as none significantly outperform naive baselines in the Leave-Drug-Out (LDO) setting. One possible explanation for this lack of generalization is that the available data may not contain a sufficiently exploitable signal. To accurately predict responses for unseen drugs, two distinct types of signal must be captured: (i) the mean drug effect, reflecting the overall potency of a compound across all cell lines, and (ii) the residual sensitivity of individual cell lines, representing deviations from this mean effect. In the CTRPv2 dataset, the central 90% of drug mean potencies range from -0.541 to 4.624 $lnIC50$, corresponding to approximately 0.6 μM to 101.5 μM (a roughly 170-fold difference), indicating that, in principle, sufficient variation in mean drug effects exists. Moreover, the mean within-drug standard deviation of the $lnIC50$ is 1.3 ($3.7\mu M$), suggesting that there are indeed cell line–specific effects. However, since the observed R^2 values are close to zero, models fail to capture even this pronounced signal in mean drug potency, let alone the more subtle, sample-specific effects.

We go on to investigate whether the failure stems from suboptimal drug representations. The main results in this study rely on Morgan fingerprints, a binary encoding derived from SMILES strings. While widely

used, this representation does not explicitly capture molecular structure or leverage chemical background knowledge. To address this, we have evaluated two alternative approaches aimed at improving molecular representation: ChemBERTaNeuralNetwork and DrugGNN. For ChemBERTaNeuralNetwork, we generate molecular embeddings using ChemBERTa [4], a transformer model pretrained on 77 million SMILES strings from PubChem [5]. Canonical SMILES are tokenized, passed through the model, and the mean of the final hidden states is used as the molecular embedding. The resulting model architecture is identical to SimpleNeuralNetwork, differing only in the use of ChemBERTa embeddings instead of fingerprints. For DrugGNN, molecular graphs are constructed from SMILES strings, where atoms are represented by concatenated one-hot encoded chemical features (atomic number, degree, charge, hydrogen count, hybridization, aromaticity, and ring membership) and bonds by their type, conjugation, and ring status. The model combines three graph convolutional layers with global mean pooling for drug encoding and a two-layer multilayer perceptron for cell line features. The resulting embeddings are concatenated and processed through fully connected layers to predict drug response. As shown in Table S1, neither ChemBERTa embeddings nor graph-based molecular representations improve performance in the LDO setting. These results indicate that, within our experimental framework, alternative molecular representations do not enhance model generalization to unseen drugs.

We therefore hypothesize that the main reason for poor generalization to new drugs is the limited number of compounds in commonly used datasets, as also argued by [6]. The theoretical space of small molecules with drug-like properties is estimated to be in the order of 10^{33} distinct compounds [7]. In contrast, CTRPv2 contains 545 compounds representing an extremely small fraction of this space. To visualize this, we generate two-dimensional UMAP embeddings from Morgan fingerprints of molecules (Figure S3). CTRPv2 compounds were filtered for single molecules with available structure. A background of 200 000 random ZINC [8] compounds provides embedding context. The UMAP was fitted on 200 000 background molecules and used to project both, background and CTRPv2 compounds. We mark the train and test molecules of the first LDO split. While the CTRPv2 compounds span most of the embedding space, their distribution is sparse relative to the dense background. This indicates that although the dataset covers a variety of scaffolds, the coverage is very shallow. Most test molecules have few structurally close neighbors in the training set. Therefore, models trained on CTRPv2 cannot rely on local chemical similarity to learn transferable structure–response relationships. When faced with a new compound in the LDO scenario, the model must extrapolate into regions of chemical space where no nearby examples exist, which, as we have shown, does not work.

In addition, structure–activity relationships in drug response are highly complex, and small chemical changes can cause large differences in activity. These so-called activity cliffs prevent reliable interpolation between structurally similar compounds. For example, Nutlin-3a and Nutlin-3b are stereoisomers with very different binding affinities for their target MDM2, despite differing by only a single stereocenter (Figure S2) [9]. Addressing activity cliffs and the vastness of the chemical space likely requires incorporating prior biological knowledge, such as perturbational gene expression profiles or mechanistic information. However, these approaches would limit a model's applicability to compounds for which such data are available, preventing predictions for entirely novel drugs.

There is also related work discussing this issue. Herbert et al. [6] performed a quantitative analysis of the failure of the LDO mode and demonstrated that remaining drug-blind performance often reflects dataset biases through pathway-level effects rather than the model's capacity. They showed that the drugs for which the global models were able to generalize stemmed from a limited set of pathways, e.g., MEK/ERK, and suggest training mechanism-of-action-specific models. Narykov et al. [10] propose a loss function that evaluates drug-wise performance instead of global performance. Guo et al. [11] further argue that drug-blind splits are still a source of data leakage and suggest splits accounting for scaffold and overall chemical similarity.

2 Supplementary Figures

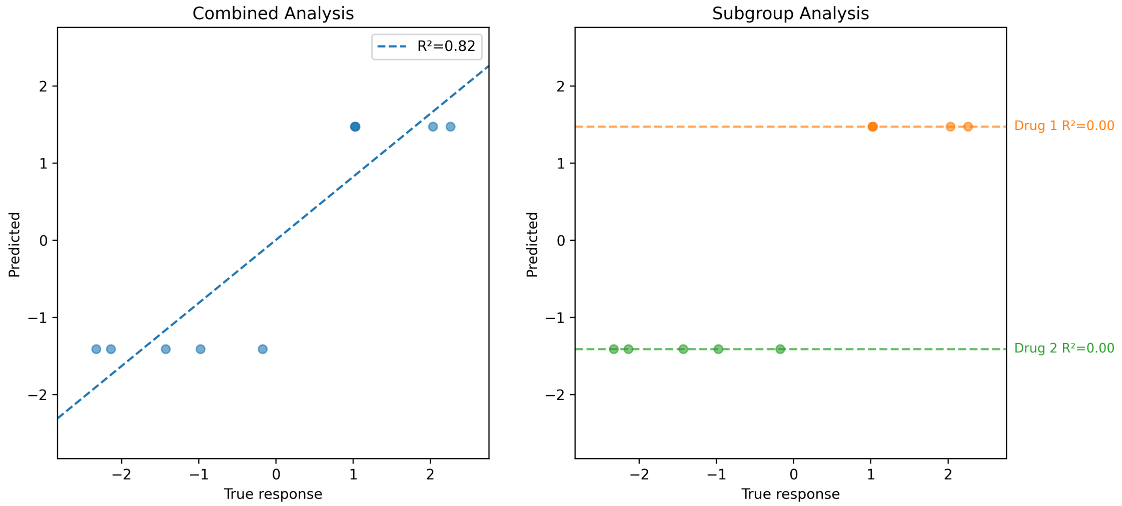

Figure S1: **Synthetic example of Simpson's paradox in drug response prediction.** We showcase the effect of Simpson's Paradox using a synthetic example with the drug response of two drugs and five cell lines, and a model that predicts just the drug mean. Left panel: All predicted versus true responses and the overall R^2, computed over $n = 8$ data points. Right panel: All predicted versus true responses, colored by drug, with drug-specific regression lines and drug-specific R^2 values annotated, computed over $n_1 = 3$ and $n_2 = 5$ data points. Simpson's Paradox: When the data is analyzed within the subgroups (drugs), the trend (high R^2) vanishes.

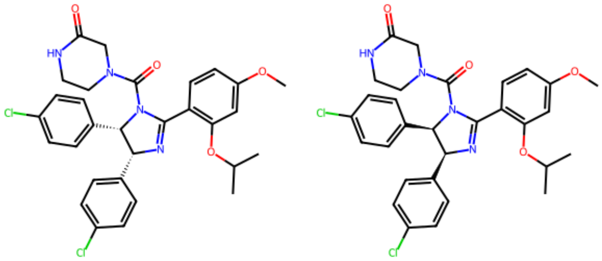

Figure S2: **Example of a drug response activity cliff.** Nutlin-3b (left) and Nutlin-3a (right) are two stereoisomers with markedly different effective binding affinities for their target MDM2 [9]

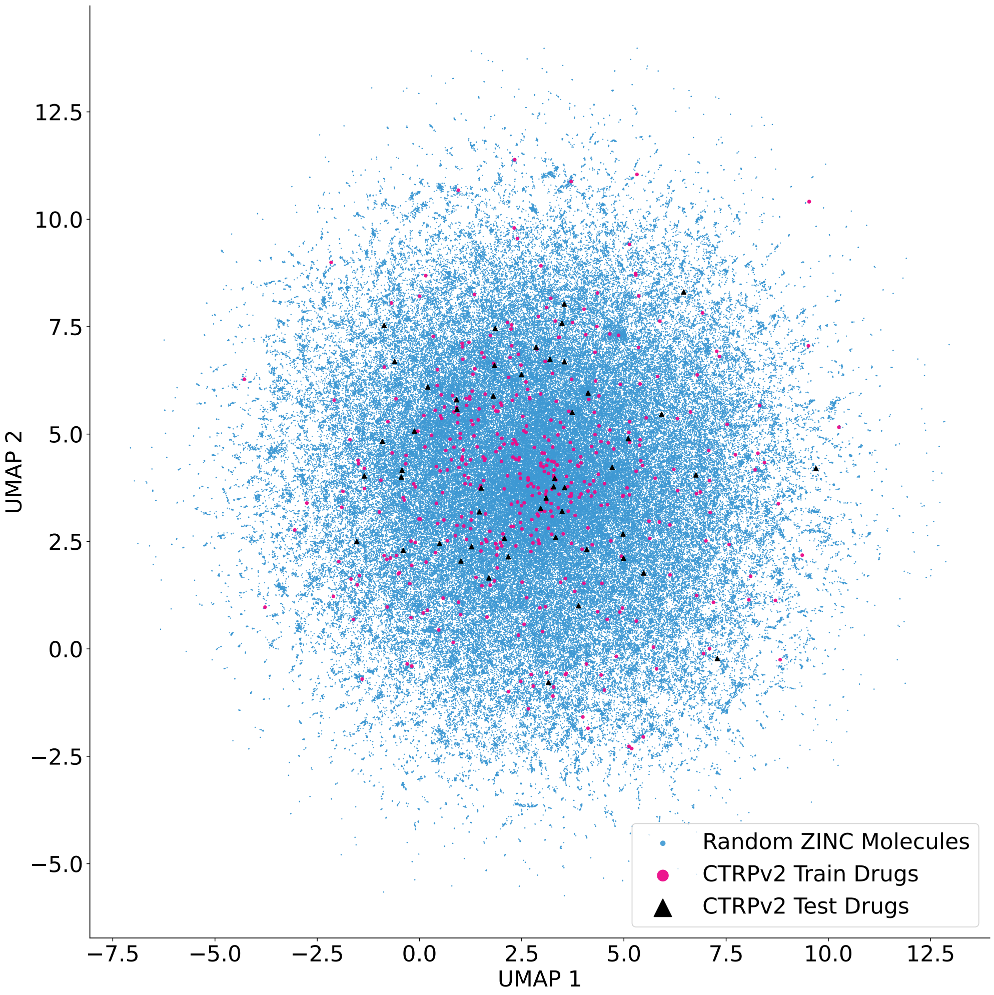

Figure S3: **UMAP of fingerprints of 200,000 random molecules from the ZINC database and CTRPv2 train and test drugs of the first cross-validation fold.** CTRPv2 train drugs are rendered in pink circles, CTRPv2 test drugs in black triangles, and the ZINC background drugs in blue small circles. While the CTRPv2 train drugs do not populate a distinct space in the UMAP, their coverage of the chemical space is still too sparse to reasonably learn drug modes of action.

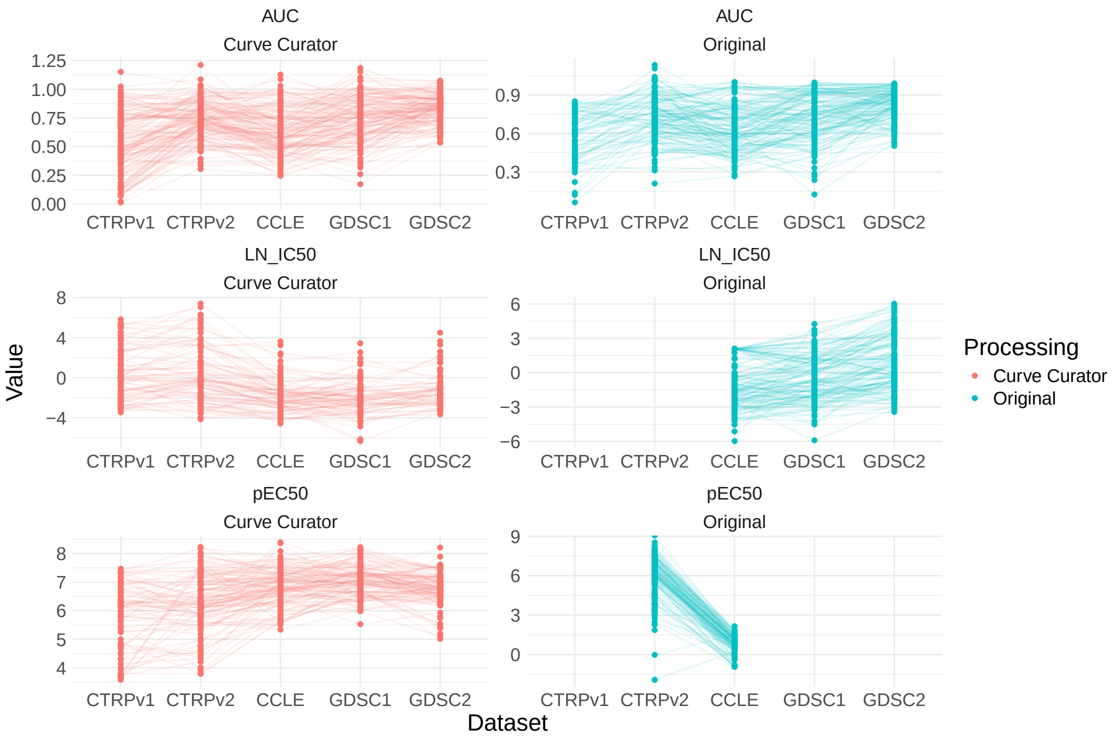

(a)

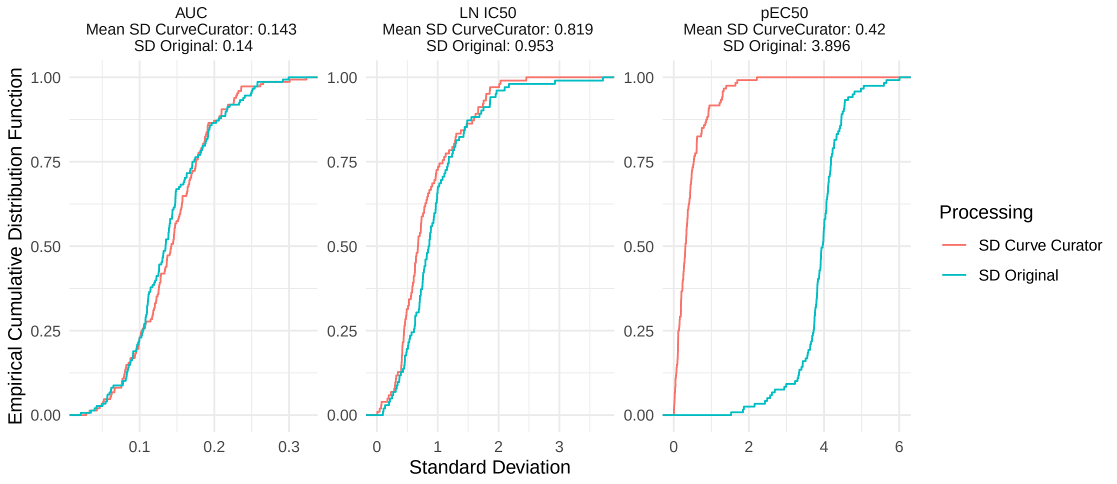

(b)

Figure S4: **Comparison of response measures in the original screen vs. reprocessed by CurveCurator. a.** One data point corresponds to one drug-cell line combination. Overall, 148 combinations occurred in all five datasets. **b.** ECDF displaying the standard deviations of the measurements of the same drug-cell line combinations between datasets. **b.** quantifies how parallel the lines in **a.** are to the x-axis: If responses roughly agree with each other between datasets, the standard deviation is small. It can be seen that through the uniform preprocessing with CurveCurator, we obtain the three metrics for all datasets.

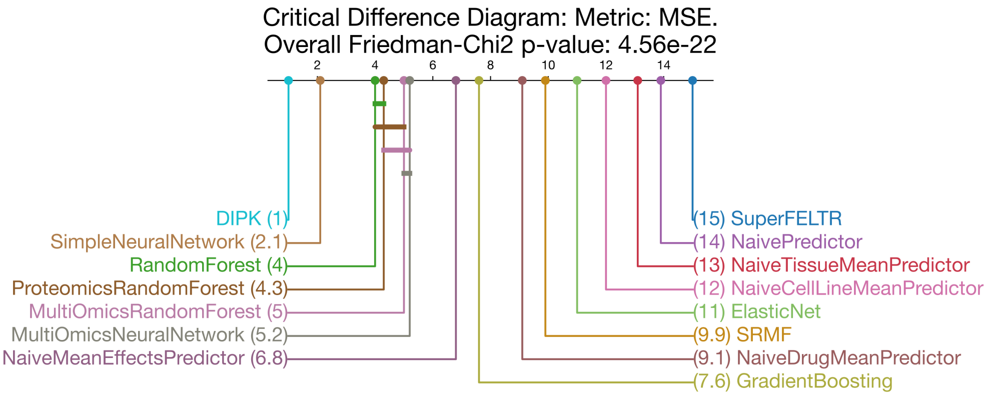

(a)

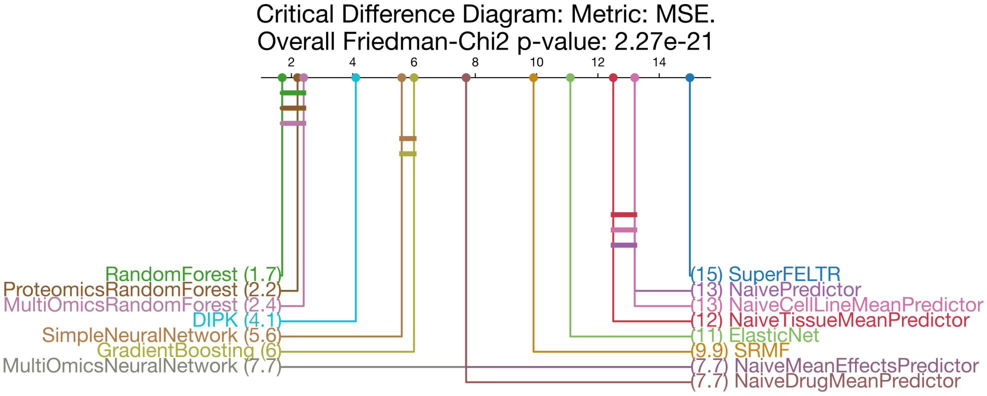

(b)

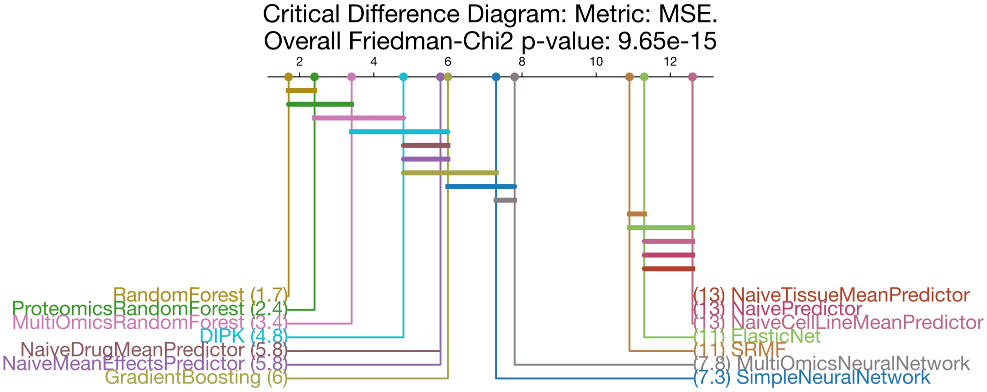

(c)

Figure S5: **Critical difference diagrams.** **a.** Leave-random-drug-cell-line-pairs-out (LPO), **b.** leave-cell-line-out (LCO), and **c.** leave-tissue-out (LTO) setting. Overall differences are first assessed using a Friedman test: number of tested models as treatments (LPO, LCO: $t = 15$; LTO: $t = 14$; LDO: $t = 11$) and 10 cross-validation folds as blocks ($n = 10$). The Friedman test assumes paired rankings across folds, without distributional assumptions. Then, the diagrams are calculated using an MSE-based ranking in the cross-validation folds. For each model, we draw a horizontal bar, connecting it to all other models from which it does not differ significantly. We assess this using a two-sided pairwise Conover test (Benjamini-Hochberg adjusted p-values < 0.05) applied to the same paired observations and introducing no additional assumptions. In the LPO and LCO setting, most differences in model performance are significant.

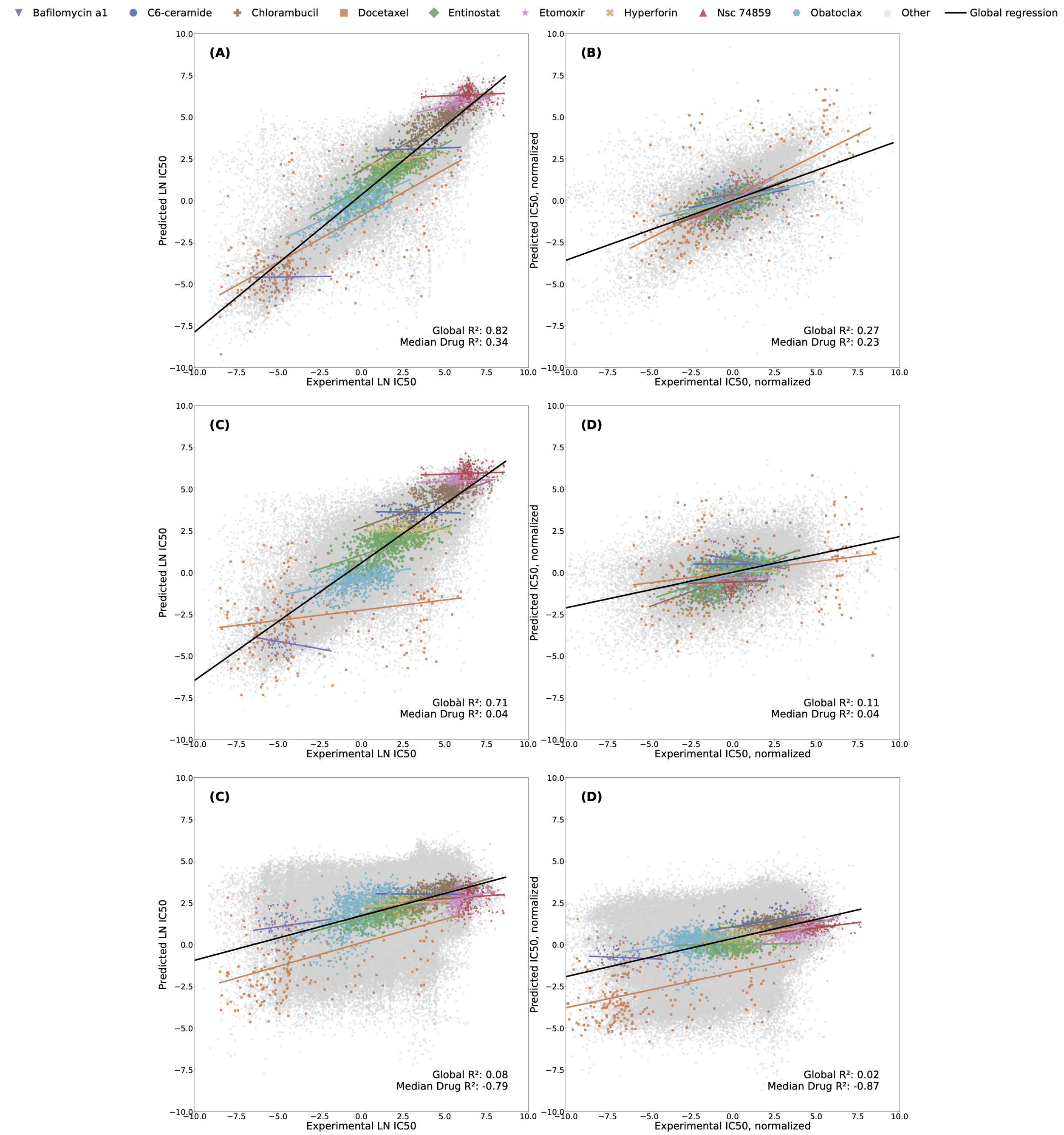

Figure S6: **Simpson's paradox in drug response prediction.** **a.** Predicted log IC50 vs. ground truth for the DIPK model under leave-random-drug-cell-line-pairs-out (LPO) cross-validation. The apparent correlation is largely driven by differences in mean drug potency. **b.** After subtracting drug and cell line mean effects, only a weak signal remains, indicating limited learning of differential response beyond remembering mean cell line and drug responses. **c.** Naive coefficients of determination are lower under leave-cell-line-out (LCO) cross-validation. **d.** However, after normalization with the drug means, the model retains more differential signal compared to LPO. **e.**, **f.** The model can not predict drug responses of unseen drugs (LDO). The global R^2's have been calculated over $\sim 220,000$ data points. The number of points underlying the per-drug R^2's varies between 3 and 793 (median: 430).

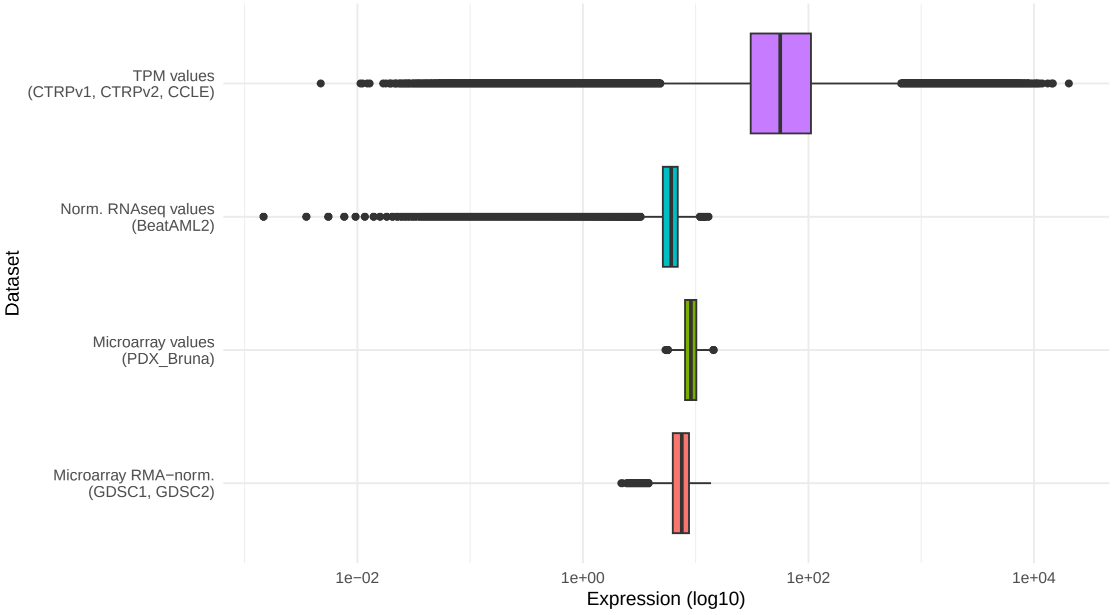

Figure S7: **Expression distribution of the microarray vs. the RNA-seq TPM expression values.** We compare the gene expression values complementing the screens of CTRPv1, CTRPv2, CCLE (purple, 275130 data points in the box), GDSC1, GDSC2 (red, 273780 data points in the box), BeatAML2 (cyan, 146610 data points in the box), and PDX_Bruna (green, 6750 data points in the box).

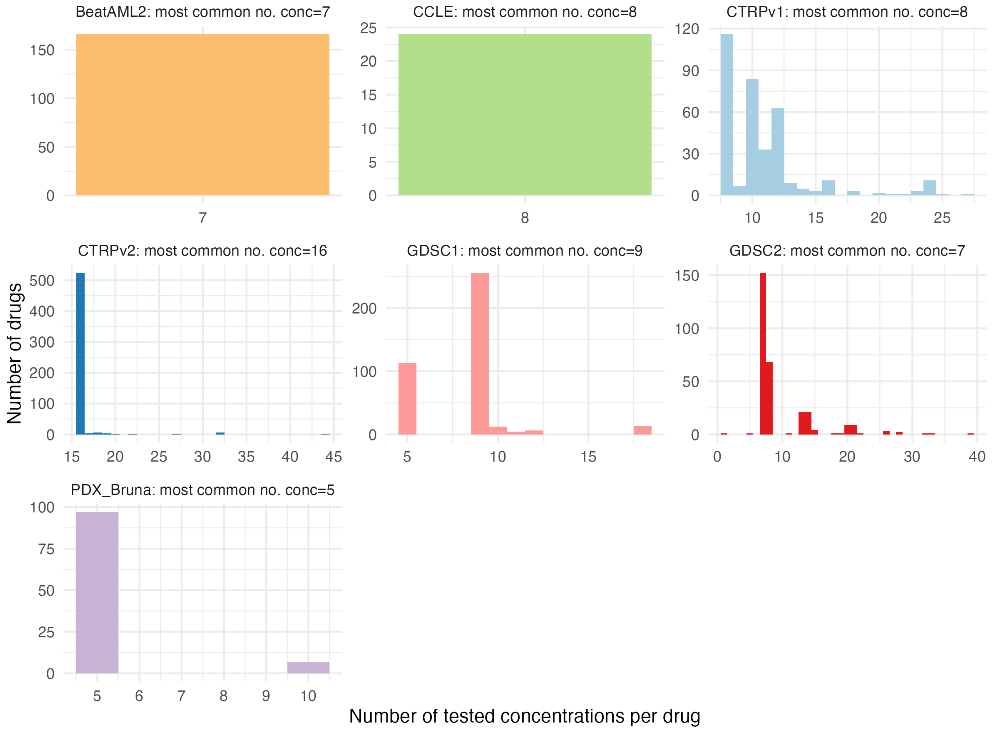

Figure S8: **Distribution of the number of measured concentrations per drug per dataset.** A curve fitted from 8 data points (as in CCLE) is not as trustworthy as a curve fitted from 16 data points (CTRPv2). More basic statistics of the drug screens are found in Supplementary Table S2.

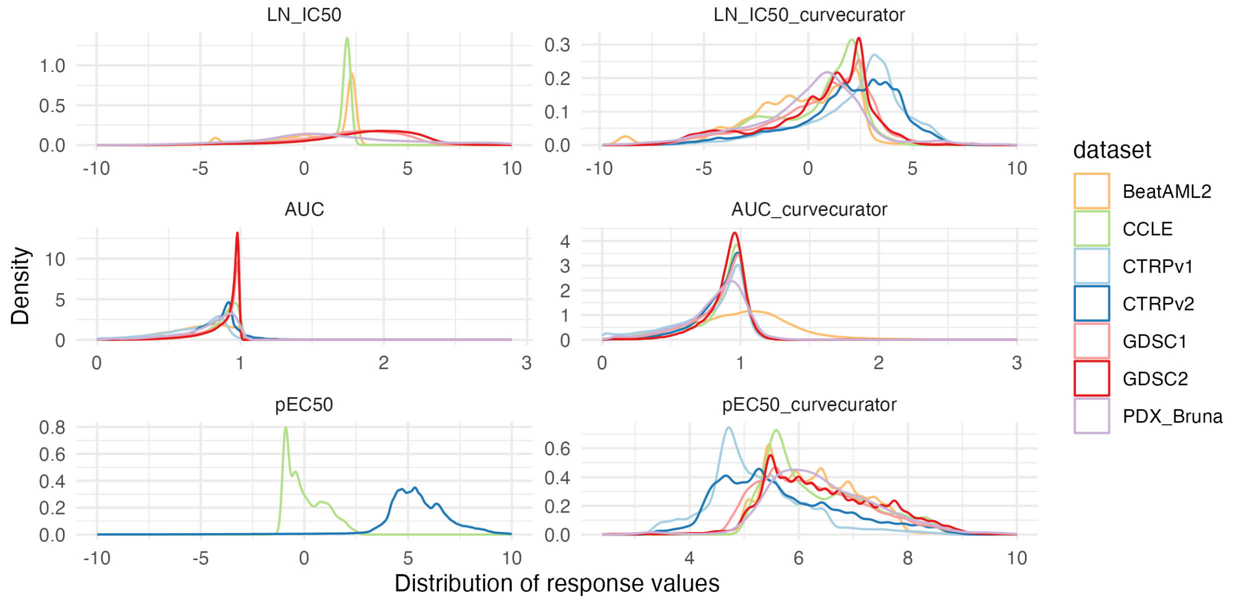

Figure S9: **Distribution of the response values originally supplied by the screens and recalculated using CurveCurator.** Extreme values ($-10 < lnIC50s < 10$, $-10 < pEC50s < 10$, $0 < AUCs < 3$) are omitted for this figure (0.83% of all values). CTRPv1 and CTRPv2 did not provide $IC50$ values, and only CCLE and CTRPv2 supplied $EC50$ values. As can be seen, refitting the curves with CurveCurator was essential, as the original measures would not have permitted attempting cross-study predictions. However, some systematic shifts are still visible (CTRPv1 and CTRPv2 have more similar distributions than GDSC1, GDSC2, and CCLE), which most likely amount to technical biases and differences between the assays. More information on the basic statistics of the drug screens is found in Supplementary Table 2.

3 Supplementary Tables

Model	MSE	R^2	R^2: normalized	Pearson	Pearson: normalized	Pearson per drug	Pearson per cell line
SimpleNeuralNetwork	**6.09±0.59**	**0.05±0.03**	**-0.02±0.03**	**0.33±0.03**	**0.26±0.03**	0.34±0.01	**0.23±0.00**
NaiveMeanEffectsPredictor	6.04±0.44	0.04±0.01	-0.03±0.01	0.26±0.01	0.00±0.00	**0.42±0.01**	–
ChemBERTaNeuralNetwork	6.33±0.55	0.01±0.03	-0.06±0.03	0.28±0.03	0.21±0.03	0.33±0.01	0.2±0.00
DrugGNN	7.08±0.67	-0.16±0.05	-0.24±0.05	0.28±0.04	0.23±0.05	0.41±0.01	0.21±0.00

Table S1: **Results of the LDO input ablation study performed on the CTRPv2 dataset.** We compare different input representations of the drug to investigate whether this is the source of the LDO failure: the SimpleNeuralNetwork uses Morgan fingerprints, the ChemBERTa precomputed ChemBERTa embeddings, and the DrugGNN uses a graph neural network to encode the drug structure. Shown are the mean ± standard errors over the 10 LDO cross-validation folds. Since none of the models is noticeably better than the others, the drug representation is most likely not the primary source of the failure to predict unseen drugs. The global measures are calculated from 174517 to 235974 data points (depending on feature availability). The data points underlying the per-drug measures vary from 3 to 857 (medians: ChemBERTaNN - 396, DrugGNN - 414, SimpleNN - 430, NaiveMeanEffectsPredictor - 462).

Dataset Name	No. unique drugs	No. unique cell lines	Nr of curves	Raw data: Nr of experiments	Doses tested per drug
CTRPv2	545	886	395 025	12 470 175	16-44
CTRPv1	354	242	60 758	1 055 049	8-27
CCLE	24	503	11 670	93 360	8
GDSC1	378	970	316 506	5 976 183	5-18
GDSC2	287	969	234 437	15 069 449	1-39
BeatAML2	166	569 (patients)	62 487	555 583	7
PDX_Bruna	104	37 (mouse passages)	2559	33 170	5-10

Table S2: **Basic statistics of the employed drug response screens.** The number of unique drugs and cell lines contained in the raw and final version of the datasets employed for this study (none were discarded), the number of rows in the raw data, and the number of doses tested per drug are displayed. The number of doses tested per drug varies by drug, i.e., some drugs have only 5 doses tested in GDSC2, while others have 18 doses (see Supplementary Figure S8 for the distribution). The distribution of the response values can be found in Supplementary Figure S9.

	**CCLE**	**CTRPv1**	**CTRPv2**	**GDSC1**	**GDSC2**	
**No. CLs in drug screen**	**503**	**242**	**886**	**970**	**969**	
**Modality**			**Overlaps**			**No. CLs in omics screen**
RNAseq gene expr. CCLE	474 (94%)	233 (96%)	820 (93%)	628 (65%)	629 (65%)	1019
Microarray gene expr. GDSC	382 (76%)	182 (75%)	590 (66%)	943 (97%)	941 (97%)	1010
RRBS methylation CCLE	415 (82%)	210 (87%)	733 (83%)	554 (57%)	555 (57%)	842
BeadChip methylation GDSC	389 (77%)	184 (76%)	596 (67%)	948 (98%)	947 (98%)	1025
CNV Cell Model Passports	369 (73%)	180 (74%)	575 (64%)	951 (98%)	951 (98%)	978
Mutation Cell Model Passports	457 (91%)	216 (89%)	759 (85%)	965 (99%)	964 (99%)	1269
DIA Proteomics	368 (73%)	177 (73%)	566 (64%)	939 (97%)	944 (97%)	949
	**BeatAML2**	**PDX_Bruna**				
**No. samples in drug screen**	**569**	**37**				
**Modality**	**Overlaps**					**No. samples in omics screen**
RNAseq gene expr. BeatAML2	487 (85%)	–				633
Microarray gene expr. PDX_Bruna	–	25 (68%)				153
CNV PDX_Bruna	–	23 (62%)				133

Table S3: **Coverage of omic screens for the different drug screens employed in this study.** The first row shows the number of unique cell lines in each of the five drug response screens included in this study. The last column shows the number of unique cell lines included in each of the omics experiments. The listed omics experiments have varying coverage of the cell lines included in the drug response screens. The absolute number of cell lines overlapping with each response screen, along with the corresponding percentages, is shown in each cell. The RNAseq data is reprocessed from [12] using the nf-core/rnaseq pipeline. The microarray data and the BeadChip methylation data was downloaded from the GDSC data portal. The RRBS methylation data were downloaded from DepMap. The DIA proteomics screen is from [13]. The two ex vivo drug screens BeatAML2 and PDX_Bruna do not have any overlap with the cell line screens or each other, hence, they are shown separately. The BeatAML2 RNAseq data is taken directly from [14] because the FASTQ files were not available. The microarray and copy number variation data for PDX_Bruna were downloaded from the figshare associated with [15].

Model / Hyperparameter	Value
NaivePredictor	None
NaiveDrugMeanPredictor	None
NaiveCellLineMeanPredictor	None
NaiveTissueMeanPredictor	None
NaiveMeanEffectsPredictor	None
ElasticNet	l1_ratio [0, 0.5, 1], alpha [1, 0.8, 0.6, 0.4, 0.2, 0.1, 5, 10, 100]
SingleDrugElasticNet	l1_ratio [0.2, 0.5, 0.9], alpha [1, 0.8, 0.6, 0.4, 0.2, 0.1, 5, 10, 100]
SingleDrugProteomicsElasticNet	l1_ratio [0.2, 0.5, 0.9], alpha [1, 0.8, 0.6, 0.4, 0.2, 0.1, 5, 10, 100]
RandomForest	n_estimators [100], max_depth [5, 10, 30], max_samples [0.2], n_jobs [-1], criterion [squared_error]
MultiOmicsRandomForest	n_estimators [100], max_depth [5, 10, 30], max_samples [0.2], n_jobs [-1], criterion [squared_error], n_components [100]
SVR	kernel [rbf], C [0.001, 0.01, 0.1, 1, 10, 100], epsilon [0.001, 0.01, 0.1, 0.5, 1], max_iter [500]
SingleDrugRandomForest	n_estimators [100], max_depth [5, 10, 30], max_samples [0.2], n_jobs [-1], criterion [squared_error]
GradientBoosting	max_iter [100], learning_rate [0.1, 0.01], max_depth [5, 10, 30]
SimpleNeuralNetwork	dropout_prob: [0.3], units_per_layer: [[32, 16, 8, 4], [128, 64, 32], [64, 64, 32], [1024, 128, 64, 16]], max_epochs: [100]
MultiOmicsNeuralNetwork	dropout_prob: [0.3], units_per_layer: [[16, 8, 4], [32, 16, 8, 4], [128, 64, 32], [64, 64, 32]], methylation_pca_components: [100], max_epochs: [100]
SRMF	K: 45, lambda_l: 0.01, lambda_d: 0, lambda_c: 0.01, max_iter: 50, seed: 1, n_features: 1036
DIPK	batch_size: [64], lr: [0.0001], heads: [2], fc_layer_num: [3], fc_layer_dim: [[256, 128, 64, 32, 16, 1]], dropout_rate: [0.3], epochs: [100], epochs_autoencoder: [100], patience: [10]
MOLIR	mini_batch: [32], h_dim1: [64, 16], h_dim2: [64, 16], h_dim3: [64, 16], learning_rate: [0.01], dropout_rate: [0.5], weight_decay: [0.0001], gamma: [0.5], epochs: [30], margin: [1.5]
SuperFELTR	mini_batch: 55, dropout_rate: [0.5], weight_decay: 0.01, out_dim_expr_encoder: 256, out_dim_mutation_encoder: 32, out_dim_cnv_encoder: 64, epochs: 30, margin: 1.0, learning_rate: 0.01

Table S4: **Hyperparameters defined per model.** Values are either scalar values or lists of tested values. If a publication reported only a single set of hyperparameters without documenting a tuning strategy (as is the case for SRMF, DIPK, and SuperFELT), we fix the configuration to the given values. For MOLI, the reported tuning strategy is random search, but the final hyperparameters are not disclosed; therefore, we varied the layer sizes while selecting reasonable values for the remaining free parameters. For the more complex models, our grid search also could not have covered the search space appropriately.

Model	MSE	R^2	R^2: normalized	Pearson	Pearson: normalized	Pearson per drug	Pearson per cell line
				LPO			
DIPK	**1.15±0.01**	**0.82±0.00**	**0.32±0.01**	**0.91±0.00**	**0.58±0.01**	**0.53±0.00**	**0.89±0.00**
SimpleNeuralNetwork	1.39±0.02	0.78±0.00	0.18±0.01	0.89±0.00	0.44±0.01	0.45±0.00	0.87±0.00
RandomForest	1.59±0.01	0.75±0.00	0.06±0.00	0.87±0.00	0.29±0.00	0.39±0.00	0.86±0.00
MultiOmicsRandomForest	1.63±0.02	0.75±0.00	0.05±0.00	0.87±0.00	0.27±0.00	0.38±0.00	0.86±0.00
MultiOmicsNeuralNetwork	1.65±0.04	0.74±0.01	0.03±0.02	0.86±0.00	0.29±0.02	0.39±0.00	0.85±0.00
NaiveMeanEffectsPredictor	1.70±0.01	0.74±0.00	0.00±0.00	0.86±0.00	0.00±0.00	0.40±0.00	0.84±0.00
GradientBoosting	1.75±0.01	0.73±0.00	-0.03±0.00	0.86±0.00	0.15±0.00	0.42±0.00	0.84±0.00
NaiveDrugMeanPredictor	2.09±0.01	0.67±0.00	-0.23±0.00	0.82±0.00	0.02±0.00	-0.21±0.00	0.84±0.00
SRMF	2.77±0.12	0.57±0.02	-0.64±0.07	0.81±0.01	0.44±0.01	0.51±0.00	0.80±0.00
ElasticNet	4.07±0.01	0.37±0.00	-1.41±0.01	0.61±0.00	0.00±0.00	0.43±0.00	0.57±0.00
NaiveCellLineMeanPredictor	6.04±0.02	0.06±0.00	-2.58±0.02	0.24±0.00	0.00±0.00	0.42±0.00	-0.19±0.00
NaiveTissueMeanPredictor	6.26±0.02	0.03±0.00	-2.71±0.02	0.16±0.00	0.02±0.00	0.29±0.00	-0.02±0.00
NaivePredictor	6.42±0.02	0.00±0.00	-2.81±0.02	0.00±0.00	0.01±0.00	0.00±0.00	-0.01±0.00
SuperFELTR	7.10±0.02	-0.10±0.00	-3.26±0.03	0.28±0.00	0.01±0.00	0.27±0.00	0.28±0.00
				LCO			
RandomForest	**1.70±0.03**	**0.74±0.01**	**0.19±0.02**	**0.86±0.00**	**0.43±0.02**	**0.32±0.00**	**0.86±0.00**
MultiOmicsRandomForest	1.74±0.02	0.73±0.00	0.16±0.02	**0.86±0.00**	0.41±0.02	0.32±0.00	**0.86±0.00**
DIPK	1.86±0.03	0.71±0.01	0.11±0.02	0.85±0.00	0.38±0.02	0.30±0.00	**0.86±0.00**
SimpleNeuralNetwork	1.94±0.03	0.70±0.01	0.07±0.02	0.84±0.00	0.35±0.02	0.26±0.00	0.85±0.00
GradientBoosting	1.98±0.04	0.69±0.01	0.05±0.01	0.84±0.00	0.29±0.01	0.27±0.00	0.84±0.00
MultiOmicsNeuralNetwork	2.08±0.04	0.68±0.01	0.00±0.03	0.83±0.00	0.28±0.02	0.26±0.00	0.83±0.00
NaiveMeanEffectsPredictor	2.09±0.04	0.68±0.00	0.00±0.00	0.82±0.00	0.00±0.00	-0.21±0.00	0.84±0.00
SRMF	2.82±0.26	0.56±0.04	-0.34±0.11	0.79±0.00	0.09±0.01	0.08±0.00	0.82±0.00
ElasticNet	5.89±0.12	0.09±0.02	-1.84±0.08	0.44±0.01	0.07±0.01	0.10±0.00	0.54±0.00
NaiveTissueMeanPredictor	6.28±0.03	0.02±0.00	-2.03±0.05	0.15±0.01	0.06±0.01	0.27±0.00	—
NaivePredictor	6.42±0.02	0.00±0.00	-2.10±0.05	0.00±0.00	0.00±0.00	-0.02±0.00	—
SuperFELTR	7.15±0.05	-0.11±0.01	-2.50±0.08	0.32±0.01	0.02±0.01	0.24±0.00	0.37±0.00

Continued on next page

Table S5: $lnIC50$ **prediction results evaluated per cross-validation (CV) fold, summarized as mean ± standard error.** There were approximately $n = 23600$ points per fold with slight variations depending on the evaluation setting. The Pearson per drug/cell line is calculated per model and test mode (CV folds combined). The underlined NaiveMeanEffectsPredictor represents the best possible performance without utilizing any features, by exploiting cell line and drug biases. The normalized metrics are derived by subtracting the predictions of the NaiveMeanEffectsPredictor from the true and predicted values and then recalculating the metric. This predictor is equivalent to the NaiveDrugMeanPredictor and the NaiveCellLineMeanPredictor for LCO/LTO and LDO, respectively. SRMF and SuperFELTR are omitted from LDO as they are not capable of predicting unseen drugs.

Model	MSE	R^2	R^2: normalized	Pearson	Pearson: normalized	Pearson per drug	Pearson per cell line
			LTO				
RandomForest	**1.80±0.06**	**0.71±0.01**	**0.07±0.01**	**0.85±0.00**	**0.29±0.02**	**0.27±0.00**	**0.85±0.00**
MultiOmicsRandomForest	1.87±0.09	0.70±0.01	0.05±0.03	0.84±0.01	0.26±0.03	0.24±0.00	**0.85±0.00**
DIPK	1.99±0.10	0.68±0.01	-0.02±0.03	0.83±0.01	0.23±0.02	0.19±0.00	0.84±0.00
GradientBoosting	2.05±0.10	0.67±0.01	-0.05±0.02	0.83±0.00	0.14±0.02	0.24±0.00	0.83±0.00
SimpleNeuralNetwork	2.12±0.08	0.66±0.01	-0.09±0.02	0.82±0.00	0.19±0.02	0.20±0.00	0.83±0.00
NaiveMeanEffectsPredictor	2.14±0.16	0.66±0.02	-0.09±0.04	0.83±0.00	0.00±0.00	-0.39±0.00	0.84±0.00
MultiOmicsNeuralNetwork	2.22±0.13	0.65±0.01	-0.13±0.03	0.81±0.01	0.14±0.02	0.21±0.00	0.82±0.00
SRMF	5.44±0.78	0.14±0.11	-1.75±0.36	0.77±0.01	0.02±0.01	-0.01±0.00	0.79±0.00
ElasticNet	6.16±0.75	0.04±0.09	-2.10±0.26	0.41±0.01	0.04±0.01	0.19±0.00	0.48±0.00
NaivePredictor	6.41±0.23	-0.02±0.01	-2.29±0.07	0.00±0.00	0.01±0.02	-0.25±0.00	—
			LDO				
MultiOmicsNeuralNetwork	**5.75±0.38**	**0.09±0.03**	**0.02±0.03**	0.34±0.03	0.26±0.04	0.33±0.00	0.28±0.00
GradientBoosting	5.86±0.52	0.08±0.03	0.01±0.03	0.29±0.06	0.21±0.05	0.36±0.00	0.24±0.00
ElasticNet	6.10±0.52	0.04±0.01	-0.03±0.01	0.24±0.02	0.12±0.02	0.32±0.00	0.16±0.00
SimpleNeuralNetwork	5.87±0.34	0.06±0.03	-0.01±0.04	0.32±0.04	0.25±0.04	0.33±0.00	0.27±0.00
DIPK	5.91±0.47	0.05±0.06	-0.02±0.06	**0.41±0.04**	**0.35±0.05**	**0.42±0.00**	**0.34±0.00**
NaiveMeanEffectsPredictor	6.03±0.53	0.05±0.01	-0.02±0.01	0.26±0.01	0.00±0.00	**0.42±0.00**	-0.18±0.00
MultiOmicsRandomForest	6.23±0.63	0.03±0.05	-0.04±0.06	0.30±0.06	0.23±0.06	0.37±0.00	0.23±0.00
NaiveTissueMeanPredictor	6.28±0.54	0.01±0.01	-0.06±0.01	0.16±0.01	0.01±0.01	0.29±0.00	-0.12±0.00
NaivePredictor	6.44±0.54	-0.02±0.00	-0.09±0.01	0.00±0.00	0.01±0.01	—	-0.12±0.00
RandomForest	6.63±0.65	-0.04±0.06	-0.12±0.06	0.26±0.05	0.20±0.05	0.36±0.00	0.20±0.00

Table S6: **CONTINUED: $lnIC50$ prediction results evaluated per cross-validation (CV) fold, summarized as mean ± standard error.** There were approximately $n = 23600$ points per fold with slight variations depending on the evaluation setting. The Pearson per drug/cell line is calculated per model and test mode (CV folds combined). The underlined NaiveMeanEffectsPredictor represents the best possible performance without utilizing any features, by exploiting cell line and drug biases. The normalized metrics are derived by subtracting the predictions of the NaiveMeanEffectsPredictor from the true and predicted values and then recalculating the metric. This predictor is equivalent to the NaiveDrugMeanPredictor and the NaiveCellLineMeanPredictor for LCO/LTO and LDO, respectively. SRMF and SuperFELTR are omitted from LDO as they are not capable of predicting unseen drugs.

Model	Runtime
DIPK	32m 51s ± 17m 21s
SimpleNeuralNetwork	23m 52s ± 9m 24s
MultiOmicsNeuralNetwork	21m 27s ± 13m 37s
ProteomicsRandomForest	9m 3s ± 2m 24s
RandomForest	7m 38s ± 6m 0s
MultiOmicsRandomForest	5m 12s ± 1m 5s
GradientBoosting	1m 43s ± 0m 55s
ElasticNet	1m 29s ± 0m 37s
SRMF	1m 11s ± 0m 27s
NaivePredictor	1m 5s ± 1m 7s
NaiveDrugMeanPredictor	0m 59s ± 1m 26s
NaiveMeanEffectsPredictor	0m 44s ± 0m 41s
NaiveCellLineMeanPredictor	0m 33s ± 0m 15s
SuperFELTR	0m 11s ± 0m 16s

Table S7: **Mean and standard deviation of model runtimes to train and test one LPO split.** Note that SuperFELTR is a single-drug model, so the run time multiplies with the number of drugs for which it is trained. Further, DIPK, SimpleNeuralNetwork, and MultiOmicsNeuralNetwork were executed on GPUs, while all other models ran on CPUs.

Model	CTRPv2	CS: CTRPv1	CS: CCLE	CS: GDSC1	CS: GDSC2	CS: BeatAML2	CS: PDX_Bruna
		LPO					
DIPK	**1.15±0.00**	4.13±0.02	5.62±0.04	8.99±0.10	7.66±0.09	21.73±0.33	14.01±0.27
SimpleNeuralNetwork	1.39±0.01	4.05±0.01	4.41±0.05	11.87±0.19	11.64±0.21	24.2±1.31	18.26±0.88
RandomForest	1.59±0.00	**3.49±0.01**	3.70±0.01	5.88±0.01	5.43±0.01	**14.61±0.03**	**10.9±0.06**
NaiveMeanEffectsPredictor	1.70±0.00	4.72±0.00	4.12±0.00	6.60±0.00	5.79±0.00	17.26±0.00	12.08±0.00
GradientBoosting	1.75±0.00	3.73±0.01	**3.43±0.02**	**5.86±0.03**	**5.35±0.02**	16.49±0.18	11.92±0.11
NaiveDrugMeanPredictor	2.09±0.00	4.25±0.00	3.78±0.00	6.45±0.00	5.82±0.00	17.26±0.00	12.08±0.00
SRMF	2.77±0.04	12.20±0.26	15.34±0.18	6658.29±3.56	5903.23±4.20	9763.1±22.58	11539.39±20.99
ElasticNet	4.07±0.00	6.43±0.04	4.43±0.02	11.19±0.27	11.37±0.27	21.75±0.17	17.46±0.16
NaiveCellLineMeanPredictor	6.04±0.01	6.52±0.00	7.19±0.01	7.84±0.00	7.92±0.00	18.46±0.00	14.75±0.00
NaivePredictor	6.42±0.01	5.99±0.00	6.70±0.01	7.70±0.00	7.98±0.00	18.46±0.00	14.75±0.00
SuperFELTR	7.10±0.01	6.95±0.00	6.46±0.01	6.92±0.00	7.95±0.00	not possible	not possible
		LCO					
DIPK	1.86±0.01	3.26±0.02	3.80±0.05	7.64±0.08	6.41±0.07	20.43±0.34	12.77±0.31
SimpleNeuralNetwork	1.94±0.01	3.44±0.01	3.62±0.04	9.16±0.21	8.64±0.19	22.77±1.16	17.43±1.23
RandomForest	**1.70±0.01**	**2.93±0.01**	3.31±0.02	**5.58±0.02**	**5.10±0.02**	**14.68±0.05**	**11.07±0.05**
NaiveMeanEffectsPredictor	2.09±0.01	3.54±0.01	3.37±0.02	6.12±0.00	5.45±0.00	17.26±0.01	12.08±0.01
GradientBoosting	1.98±0.01	3.19±0.01	**3.22±0.03**	5.63±0.04	5.09±0.04	16.24±0.23	11.67±0.16
SRMF	2.82±0.08	7.78±0.28	5.16±0.12	12530.41±15.87	10880.02±15.31	9799.45±54.91	11553.32±67.80
ElasticNet	5.89±0.04	6.29±0.06	6.16±0.05	11.59±0.62	11.77±0.62	22.07±0.59	17.76±0.51
NaivePredictor	6.42±0.01	6.18±0.02	7.68±0.02	7.90±0.00	8.46±0.00	18.46±0.01	14.75±0.01
SuperFELTR	7.15±0.02	7.01±0.01	6.38±0.01	6.89±0.00	7.92±0.01	not possible	not possible
		LDO					
DIPK	5.91±0.15	6.21±0.05	4.80±0.14	9.21±0.11	8.00±0.13	18.67±0.95	13.75±0.56
SimpleNeuralNetwork	**5.87±0.11**	5.90±0.02	**3.74±0.15**	7.29±0.15	7.60±0.17	18.04±3.08	17.64±1.56
RandomForest	6.63±0.21	6.01±0.08	5.59±0.25	6.84±0.05	6.59±0.09	12.17±0.65	**12.11±0.39**
NaiveMeanEffectsPredictor	6.03±0.17	6.78±0.01	5.41±0.23	7.40±0.02	6.98±0.04	13.73±0.57	14.26±0.20
GradientBoosting	5.86±0.16	**5.63±0.03**	4.57±0.20	**6.45±0.03**	**6.15±0.05**	13.42±0.74	12.99±0.40
ElasticNet	6.10±0.16	5.90±0.02	4.82±0.25	6.54±0.02	6.34±0.03	**11.29±0.60**	13.94±0.29
NaivePredictor	6.44±0.17	6.36±0.01	5.18±0.23	7.26±0.02	7.04±0.04	13.73±0.57	14.26±0.20

Table S8: **MSE values evaluated per cross-validation fold, summarized as mean ± standard errors for all cross-study predictions.** CS: cross-study. All models are trained on CTRPv2. Test sets include the test folds of CTRPv2 (approximately $n_0 = 23600$ data points per fold), CTRPv1 (approximately $n_1^{LPO} = 26000$, $n_1^{LCO} = 7500$, $n_1^{LDO} = 16000$ points), CCLE (approximately $n_2^{LPO} = 4200$, $n_2^{LCO} = 1400$, $n_2^{LDO} = 2400$ points), GDSC1 (approximately $n_3^{LPO} = 142000$, $n_3^{LCO} = 74000$, $n_3^{LDO} = 116000$ points), and GDSC2 (approximately $n_4^{LPO} = 78000$, $n_4^{LCO} = 42000$, $n_4^{LDO} = 60000$ points). The trained models further predict the ex vivo responses of the BeatAML2 (approximately $n_5^{LPO} = n_5^{LCO} = 22500$, $n_5^{LDO} = 10000$ points) and the PDX_Bruna (approximately $n_6^{LPO} = n_6^{LCO} = 1500$, $n_6^{LDO} = 800$ points) dataset. Drug-cell line combination counts differ from those in Figure 1 for two reasons: LN_IC50 values deemed unrealistic were set to NA during preprocessing, and some cell lines or drugs overlapped with the CTRPv2 training fold, depending on the setting.

Model	CTRPv2	CS: CTRPv1	CS: CCLE	CS: GDSC1	CS: GDSC2	CS: BeatAML2	CS: PDX_Bruna
		LCO lnIC50: MSE					
Random Forest	1.70±0.03	2.93±0.02	3.31±0.05	5.58±0.05	5.10±0.04	14.68±0.05	11.07±0.05
Naive Mean Effects Predictor	2.09±0.11	3.54±0.11	3.37±0.22	6.12±0.02	5.45±0.03	17.26±0.01	12.08±0.01
		LCO AUC: MSE					
RandomForest	0.012±0.000	0.065±0.001	0.022±0.000	0.049±0.000	0.039±0.000	0.228±0.001	0.051±0.000
NaiveMeanEffectsPredictor	0.015±0.001	0.069±0.000	0.023±0.000	0.041±0.000	0.024±0.000	0.201±0.000	0.053±0.000
		LCO pEC50: MSE					
RandomForest	0.68±0.01	0.83±0.00	1.30±0.02	1.13±0.01	1.04±0.01	38.27±0.08	36.15±0.05
NaiveMeanEffectsPredictor	0.70±0.01	0.78±0.01	1.35±0.01	1.29±0.00	1.52±0.00	29.1±0.01	28.31±0.01
		LCO lnIC50: Pearson					
RandomForest	0.86±0.00	0.72±0.00	0.74±0.00	0.42±0.01	0.51±0.01	0.17±0.00	0.44±0.00
NaiveMeanEffectsPredictor	0.82±0.00	0.65±0.00	0.71±0.00	0.44±0.00	0.55±0.00	0.18±0.00	0.39±0.00
		LCO AUC: Pearson					
RandomForest	0.8±0.00	0.48±0.00	0.75±0.00	0.25±0.00	0.31±0.01	0.29±0.00	0.29±0.00
NaiveMeanEffectsPredictor	0.75±0.00	0.38±0.00	0.73±0.00	0.28±0.00	0.33±0.00	0.24±0.00	0.19±0.00
		LCO pEC50: Pearson					
RandomForest	0.68±0.00	0.53±0.01	0.24±0.01	0.15±0.01	0.24±0.01	0.07±0.00	0.29±0.01
NaiveMeanEffectsPredictor	0.67±0.00	0.53±0.00	0.24±0.00	0.29±0.00	0.34±0.00	0.14±0.00	0.40±0.00
		LCO lnIC50: R^2					
RandomForest	0.74±0.01	0.52±0.00	0.36±0.01	0.11±0.01	0.22±0.01	-0.08±0.00	0.16±0.00
NaiveMeanEffectsPredictor	0.68±0.00	0.41±0.00	0.36±0.00	0.02±0.00	0.17±0.00	-0.25±0.00	-0.02±0.00
		LCO AUC: R^2					
RandomForest	0.63±0.00	0.06±0.00	0.52±0.00	-0.12±0.00	-0.64±0.02	-0.19±0.00	-0.01±0.01
NaiveMeanEffectsPredictor	0.56±0.01	0.00±0.00	0.49±0.00	0.07±0.00	-0.01±0.00	-0.07±0.00	-0.03±0.00
		LCO pEC50: R^2					
RandomForest	0.46±0.00	0.03±0.01	-0.59±0.02	-0.06±0.01	0.03±0.01	-60.37±0.12	-34.13±0.05
NaiveMeanEffectsPredictor	0.45±0.00	0.09±0.01	-0.68±0.01	-0.21±0.00	-0.42±0.00	-45.76±0.01	-27.84±0.01

Table S9: **Metrics for cross-study predictions with other response measures (all re-computed with CurveCurator), mean ± standard error.** Note that the MSEs are not comparable because of different scales of lnIC50, pEC50, and AUC. CS: cross-study. For the number of data points underlying the LN_IC50 metrics, please refer to the n_i^{LCO} numbers of Table S8. Data points underlying the global performance measures for the AUC experiments: CTRPv2 - ∼ 39500 data points per fold, CTRPv1 - ∼ 12000, CCLE - ∼ 2300, GDSC1 - ∼ 146000, GDSC2 - ∼ 106000, BeatAML2 - ∼ 62000, PDX_Bruna - ∼ 2500. For pEC50: CTRPv2 - ∼ 34700 data points per fold, CTRPv1 - ∼ 9100, CCLE - ∼ 1900, GDSC1 - ∼ 115000, GDSC2 - ∼ 85000, BeatAML2 - ∼ 46400, PDX_Bruna - ∼ 2000.

Setting	Δ MSE	ΔR^2	Δ Pearson
LPO Multi-OMICS Random Forest			
CNV (Perm.)	0.00±0.00	0.00±0.00	0.00±0.00
CNV (Inv.)	0.02±0.00	0.00±0.00	0.00±0.00
**Expression (Perm.)**	0.15±0.00	-0.02±0.00	-0.01±0.00
Expression (Inv.)	0.14±0.00	-0.02±0.00	-0.01±0.00
Methylation (Perm.)	0.01±0.00	0.00±0.00	0.00±0.00
Methylation (Inv.)	0.01±0.00	0.00±0.00	0.00±0.00
Mutation (Perm.)	0.00±0.00	0.00±0.00	0.00±0.00
Mutation (Inv.)	0.02±0.00	0.00±0.00	0.00±0.00
Fingerprints (Perm.)	0.09±0.01	-0.01±0.00	-0.01±0.00
Fingerprints (Inv.)	0.02±0.00	0.00±0.00	0.00±0.00
LPO DIPK			
BIONIC (Perm.)	0.01±0.01	0.00±0.00	0.00±0.00
BIONIC (Inv.)	0.00±0.01	0.00±0.00	0.00±0.00
Expression (Perm.)	0.05±0.01	-0.01±0.00	0.00±0.00
Expression (Inv.)	-0.04±0.01	0.01±0.00	0.00±0.00
MolGNet (Perm.)	0.05±0.01	-0.01±0.00	0.00±0.00
**MolGNet (Inv.)**	0.06±0.01	-0.01±0.00	-0.01±0.00
LCO Multi-OMICS Random Forest			
CNV (Perm.)	0.00±0.00	0.00±0.00	0.00±0.00
CNV (Inv.)	0.03±0.00	0.00±0.00	0.00±0.00
**Expression (Perm.)**	0.20±0.02	-0.03±0.00	-0.02±0.00
Expression (Inv.)	0.18±0.02	-0.03±0.00	-0.02±0.00
Methylation (Perm.)	0.01±0.00	0.00±0.00	0.00±0.00
Methylation (Inv.)	0.01±0.00	0.00±0.01	0.00±0.00
Mutation (Perm.)	0.00±0.00	0.00±0.00	0.00±0.00
Mutation (Inv.)	0.03±0.01	0.00±0.00	0.00±0.00
Fingerprints (Perm.)	0.04±0.01	-0.01±0.00	0.00±0.00
Fingerprints (Inv.)	-0.01±0.01	0.00±0.00	0.00±0.00
LCO DIPK			
BIONIC (Perm.)	0.01±0.02	0.00±0.00	0.00±0.00
BIONIC (Inv.)	-0.01±0.03	0.00±0.00	0.00±0.00
Expression (Perm.)	0.18±0.02	-0.03±0.00	-0.02±0.00
**Expression (Inv.)**	0.20±0.03	-0.03±0.00	-0.01±0.00
MolGNet (Perm.)	0.03±0.03	0.00±0.00	0.00±0.00
MolGNet (Inv.)	0.00±0.02	0.00±0.00	0.00±0.00
LDO Multi-OMICS Random Forest			
CNV (Perm.)	0.04±0.04	-0.01±0.01	0.00±0.01
CNV (Inv.)	0.04±0.04	-0.01±0.01	0.00±0.01
Expression (Perm.)	0.08±0.06	-0.01±0.01	-0.01±0.01
Expression (Inv.)	0.19±0.05	-0.03±0.01	-0.03±0.01
Methylation (Perm.)	0.06±0.05	-0.01±0.01	0.00±0.01
Methylation (Inv.)	0.01±0.05	0.00±0.01	0.01±0.01
Mutation (Perm.)	0.07±0.04	-0.01±0.01	-0.01±0.01
Mutation (Inv.)	0.10±0.04	-0.02±0.02	-0.01±0.01
**Fingerprints (Perm.)**	0.95±0.33	-0.16±0.07	-0.17±0.05
Fingerprints (Inv.)	0.38±0.30	-0.08±0.06	-0.09±0.05
LDO DIPK			
BIONIC (Perm.)	-0.11±0.12	0.02±0.02	-0.01±0.01
BIONIC (Inv.)	-0.22±0.14	0.04±0.02	0.01±0.01
Expression (Perm.)	-0.15±0.11	0.03±0.02	0.01±0.01
Expression (Inv.)	-0.22±0.20	0.03±0.03	0.00±0.02
**MolGNet (Perm.)**	2.61±0.38	-0.39±0.05	-0.31±0.04
MolGNet (Inv.)	2.07±0.33	-0.33±0.06	-0.35±0.04

Table S10: **Ablation study results of the Multi-OMICS Random Forest and DIPK.** One OMIC has been randomized at a time. Perm: a drug/cell line has received a random feature of another drug/cell line. Inv: summary-statistic-invariant randomization. The performance measures of the unperturbed models are subtracted from those of the ablated models for each of the $k = 10$ cross-validation folds, and the results are then averaged; negative values indicate that the perturbation decreased model performance. The number of points per fold varies between ~ 9600 and ~ 22000.

Model	MSE	R^2	R^2: normalized	Pearson	Pearson: normalized	Pearson per drug	Pearson per cell line
			LPO				
Random Forest	1.58±0.04	0.75±0.01	0.06±0.01	0.87±0.00	0.30±0.01	0.40±0.26	0.86±0.08
Proteomics Random Forest	1.61±0.01	0.75±0.00	0.05±0.00	0.87±0.00	0.27±0.00	0.38±0.00	0.86±0.00
Naive Mean Effects Predictor	1.70±0.03	0.74±0.01	0.00±0.00	0.86±0.00	0.00±0.00	0.40±0.25	0.84±0.09
			LCO				
Random Forest	1.70±0.11	0.74±0.02	0.19±0.05	0.86±0.01	0.43±0.05	0.33±0.25	0.86±0.08
Proteomics Random Forest	1.73±0.05	0.73±0.01	0.16±0.01	0.86±0.00	0.40±0.02	0.30±0.00	0.86±0.00
Naive Mean Effects Predictor	2.09±0.11	0.68±0.02	0.00±0.00	0.82±0.01	0.00±0.00	-0.21±0.19	0.84±0.08
			LTO				
Random Forest	1.82±0.07	0.71±0.01	0.06±0.02	0.85±0.01	0.27±0.03	0.26±0.00	0.85±0.00
Proteomics Random Forest	1.83±0.07	0.71±0.01	0.06±0.02	0.85±0.01	0.26±0.03	0.26±0.00	0.85±0.00
Naive Mean Effects Predictor	2.14±0.16	0.66±0.02	-0.09±0.04	0.83±0.00	0.00±0.00	-0.39±0.00	0.84±0.00
			LDO				
Naive Mean Effects Predictor	6.03±1.67	0.05±0.02	-0.02±0.02	0.26±0.03	0.00±0.00	0.42±0.23	-0.18±0.07
Proteomics Random Forest	6.14±0.49	0.03±0.06	-0.04±0.06	0.32±0.05	0.26±0.05	0.37±0.00	0.25±0.00
Random Forest	6.58±2.03	-0.04±0.19	-0.11±0.20	0.27±0.16	0.21±0.16	0.36±0.26	0.21±0.07

Table S11: **Predictive performance of the Proteomics Random Forest.** Performance metrics are reported as Mean ± Standard Error (SE) calculated across 10-fold cross-validation splits ($k = 10$). The model was evaluated on $N = 153,005$ drug-sensitivity measurements with $n = 566$ unique cell lines and 545 unique drugs. The underlined values indicate the baseline performance of the NaiveMeanEffectsPredictor.

Hallmark	Self-assigned score (0-5)	Reason
Data Relevance and Actionability	4 in terms of commonly used data, but ultimately model-specific	All data is accessible via Zenodo. We include PDX_Bruna and BeatAML2 as datasets for testing clinically more realistic data but we do not have a patient dataset with, e.g., RECIST drug response. All input data could be obtained in a clinical setting (drug structures, RNAseq, mutation, methylation, copy number variation, proteomics). Ultimately, however, the models themselves are responsible for the complexity of their data input. In any case, there should be no data leakage from standardization or joined processing.
Expressive Architecture	Model-specific; cannot be properly assessed	We have implemented various models on the bias-variance tradeoff scale: some models are too simple to (theoretically) capture complex biological relationships, while some have many learnable parameters. However, since the models are currently not learning biologically relevant relationships, this cannot be properly assessed.
Standardized Benchmarking	5	This is the primary purpose of DrEval. We implement various baselines, have processed and provided multiple datasets, and implement consistent data splits, multiple metrics, and a uniform protocol for hyperparameter optimization.
Demonstrated Generalizability	3	We only assess cross-study prediction from one cell-line-based screen to others and to two ex vivo datasets, one of them directly cultivated from patient cells, one from PDX. As demonstrated, most models struggle with this kind of generalization already. We have not implemented a way to easily switch from predicting the response metric to predicting a clinically relevant metric, such as the RECIST criteria, progression-free survival, or overall survival. We do not include transfer learning approaches yet.
Mechanistic Interpretability	0	Currently, we have not implemented a model-agnostic post-hoc diagnostic that is automatically run at the end of the pipeline, such as SHAP or LIME. We have also not included models with explainability modules, such as LRP or uncertainty measures.
Accessibility and Reproducibility	5	This is also a primary goal of DrEval. All models, code, and data are accessible. Through the pipeline, all results are easily reproducible with a single command.
Fairness	0; cannot be properly assessed	So far, we have not quantified whether models exhibit predictive biases with respect to patient populations (including race/ethnicity, sex, age, social determinants, and diseases). While such analyses are important, they remain a future goal because current models fail to generalize reliably. This makes it difficult to disentangle bias from overall poor performance.

Table S12: **Study limitations structured by the categories proposed by [16].** Singhal et al. introduce seven hallmarks in their publication "The Hallmarks of Predictive Oncology" by which developers can assess whether their predictive oncology model has the requirements of being considered for clinical practice. Ideally, the scores ($0-5$, 5 being the best score) should be assigned by external developers trying to reproduce the model results. Some of the hallmarks are model-specific by design and, therefore, their corresponding scores cannot accurately reflect the quality of the entire pipeline.

Test mode	df	Q	p-value	Kendall's W
LPO	14	135.9300	4.56e-22	0.9709
LCO	14	132.4129	2.27e-21	0.9458
LTO	13	96.0451	9.65e-15	0.7388
LDO	10	6.8182	7.42e-01	0.0682

Table S13: **Statistical test details for the Friedman-Chi2 tests.** For the four conducted Friedman-Chi2 tests, we report the degrees of freedom (df, number of compared models -1), the test statistic (Q), the p-value ($P(\chi^2_{k-1} \geq Q)$: always one-sided), and Kendall's W, a measure of effect size ($Q/(n \times (k-1))$ with n =number of cross-validation folds, i.e., 10, k =number of models). The effect is considered small for $W > 0.1$, moderate for $W > 0.3$, and strong for $W > 0.5$.

	DIPK	ENet	GB	MO-NN	MO-RF	CL Mean	Drug Mean	Mean Eff.	Naive Pred	Tissue Mean	Prot. RF	RF	SRMF	Simple-NN	SuperFELTR
Benjamini-Hochberg adjusted p-values															
DIPK	1.0e+00	7.3e-54	2.8e-36	4.5e-21	9.1e-20	3.0e-58	1.2e-44	1.9e-31	1.3e-65	1.4e-62	4.0e-15	3.3e-13	9.9e-49	3.5e-03	3.0e-69
Elastic Net	7.3e-54	1.0e+00	8.9e-16	1.9e-31	1.2e-32	7.6e-03	9.5e-07	4.5e-21	1.4e-12	8.2e-08	7.1e-37	1.3e-38	3.5e-03	9.9e-49	9.1e-20
Gradient Boosting	2.8e-36	8.9e-16	1.0e+00	1.6e-09	1.0e-10	2.1e-22	8.4e-05	3.1e-02	1.7e-34	1.5e-29	4.0e-15	4.2e-17	6.2e-09	1.5e-29	7.4e-41
Multi Omics Neural Network	4.5e-21	1.9e-31	1.6e-09	1.0e+00	5.9e-01	1.9e-37	4.2e-19	2.9e-05	9.5e-48	1.4e-43	1.6e-02	1.5e-03	2.1e-24	7.7e-14	5.1e-53
Multi Omics Random Forest	9.1e-20	1.2e-32	1.0e-10	5.9e-01	1.0e+00	1.3e-38	2.1e-20	3.1e-06	9.9e-49	1.2e-44	5.9e-02	7.6e-03	1.1e-25	1.4e-12	7.3e-54
Naive Cell Line Mean Predictor	3.0e-58	7.6e-03	2.1e-22	1.9e-37	1.3e-38	1.0e+00	1.4e-12	1.3e-27	9.5e-07	3.5e-03	1.6e-42	4.0e-44	8.2e-08	1.8e-53	3.3e-13
Naive Drug Mean Predictor	1.2e-44	9.5e-07	8.4e-05	4.2e-19	2.1e-20	1.4e-12	1.0e+00	6.2e-09	4.8e-25	9.1e-20	4.8e-25	5.4e-27	3.1e-02	1.3e-38	4.8e-32
Naive Mean Effects Predictor	1.9e-31	4.5e-21	3.1e-02	2.9e-05	3.1e-06	1.3e-27	6.2e-09	1.0e+00	3.7e-39	1.7e-34	4.2e-10	6.0e-12	7.7e-14	2.1e-24	3.8e-45
Naive Predictor	1.3e-65	1.4e-12	1.7e-34	9.5e-48	9.9e-49	9.5e-07	4.8e-25	3.7e-39	1.0e+00	3.1e-02	4.3e-52	1.8e-53	9.1e-20	1.9e-61	3.5e-03
Naive Tissue Mean Predictor	1.4e-62	8.2e-08	1.5e-29	1.4e-43	1.2e-44	3.5e-03	9.1e-20	1.7e-34	3.1e-02	1.0e+00	3.0e-48	1.2e-49	1.8e-14	3.0e-58	9.5e-07
Proteomics Random Forest	4.0e-15	7.1e-37	4.0e-15	1.6e-02	5.9e-02	1.6e-42	4.8e-25	4.2e-10	4.3e-52	3.0e-48	1.0e+00	4.2e-01	3.5e-30	2.3e-08	5.6e-57
Random Forest	3.3e-13	1.3e-38	4.2e-17	1.5e-03	7.6e-03	4.0e-44	5.4e-27	6.0e-12	1.8e-53	1.2e-49	4.2e-01	1.0e+00	4.8e-32	9.5e-07	3.0e-58
SRMF	9.9e-49	3.5e-03	6.2e-09	2.1e-24	1.1e-25	8.2e-08	3.1e-02	7.7e-14	9.1e-20	1.8e-14	3.5e-30	4.8e-32	1.0e+00	4.6e-43	5.4e-27
Simple Neural Network	3.5e-03	9.9e-49	1.5e-29	7.7e-14	1.4e-12	1.8e-53	1.3e-38	2.1e-24	1.9e-61	3.0e-58	2.3e-08	9.5e-07	4.6e-43	1.0e+00	1.3e-65
SuperFELTR	3.0e-69	9.1e-20	7.4e-41	5.1e-53	7.3e-54	3.3e-13	4.8e-32	3.8e-45	3.5e-03	9.5e-07	5.6e-57	3.0e-58	5.4e-27	1.3e-65	1.0e+00
Test statistic: t-statistic, df=126															
DIPK	0.0	27.354	18.053	11.489	10.941	30.089	22.156	15.865	35.286	33.098	9.027	8.206	24.345	3.009	38.295
Elastic Net	27.354	0.0	9.3	15.865	16.412	2.735	5.197	11.489	7.933	5.744	18.327	19.148	3.009	24.345	10.941
Gradient Boosting	18.053	9.3	0.0	6.565	7.112	12.036	4.103	2.188	17.233	15.044	9.027	9.847	6.291	15.044	20.242
Multi Omics Neural Network	11.489	15.865	6.565	0.0	0.547	18.6	10.668	4.377	23.798	21.609	2.462	3.282	12.856	8.48	26.807
Multi Omics Random Forest	10.941	16.412	7.112	0.547	0.0	19.148	11.215	4.924	24.345	22.156	1.915	2.735	13.403	7.933	27.354
Naive Cell Line Mean Predictor	30.089	2.735	12.036	18.6	19.148	0.0	7.933	14.224	5.197	3.009	21.062	21.883	5.744	27.08	8.206
Naive Drug Mean Predictor	22.156	5.197	4.103	10.668	11.215	7.933	0.0	6.291	13.13	10.941	13.13	13.95	2.188	19.148	16.139
Naive Mean Effects Predictor	15.865	11.489	2.188	4.377	4.924	14.224	6.291	0.0	19.421	17.233	6.838	7.659	8.48	12.856	22.43
Naive Predictor	35.286	7.933	17.233	23.798	24.345	5.197	13.13	19.421	0.0	2.188	26.259	27.08	10.941	32.277	3.009
Naive Tissue Mean Predictor	33.098	5.744	15.044	21.609	22.156	3.009	10.941	17.233	2.188	0.0	24.071	24.892	8.753	30.089	5.197
Proteomics Random Forest	9.027	18.327	9.027	2.462	1.915	21.062	13.13	6.838	26.259	24.071	0.0	0.821	15.318	6.018	29.268
Random Forest	8.206	19.148	9.847	3.282	2.735	21.883	13.95	7.659	27.08	24.892	0.821	0.0	16.139	5.197	30.089
SRMF	24.345	3.009	6.291	12.856	13.403	5.744	2.188	8.48	10.941	8.753	15.318	16.139	0.0	21.336	13.95
Simple Neural Network	3.009	24.345	15.044	8.48	7.933	27.08	19.148	12.856	32.277	30.089	6.018	5.197	21.336	0.0	35.286
SuperFELTR	38.295	10.941	20.242	26.807	27.354	8.206	16.139	22.43	3.009	5.197	29.268	30.089	13.95	35.286	0.0

Table S14: **Statistical test details for the LPO pairwise post-hoc Conover tests.** For all tests, the degrees of freedom are calculated as $nk - k - n + 1 = 126$, where n =number of cross-validation folds, i.e., 10, and k =number of models, i.e., 15. All p-values are multiple testing-adjusted with Benjamini-Hochberg correction. They are derived from the t-statistics given. The post-hoc Conover test is always two-sided.

	DIPK	ENet	GB	MO-NN	MO-RF	CL Mean	Drug Mean	Mean Eff.	Naive Pred	Tissue Mean	Prot. RF	RF	SRMF	Simple-NN	SuperFELTR
							Benjamini-Hochberg adjusted p-values								
DIPK	1.0e+00	6.2e-31	4.8e-05	7.4e-13	2.4e-04	5.5e-41	7.4e-13	7.4e-13	5.5e-41	9.1e-38	4.8e-05	4.8e-07	1.3e-24	1.1e-03	1.5e-48
Elastic Net	6.2e-31	1.0e+00	6.3e-21	7.7e-12	3.5e-39	8.0e-06	7.7e-12	7.7e-12	8.0e-06	2.3e-03	4.2e-40	2.6e-42	8.9e-03	4.4e-23	2.0e-14
Gradient Boosting	4.8e-05	6.3e-21	1.0e+00	2.4e-04	7.4e-13	5.9e-32	2.4e-04	2.4e-04	5.9e-32	2.5e-28	6.8e-14	1.4e-16	2.0e-14	3.9e-01	1.5e-40
Multi Omics Neural Network	7.4e-13	7.7e-12	2.4e-04	1.0e+00	5.2e-22	4.4e-23	1.0e+00	1.0e+00	4.4e-23	2.7e-19	4.4e-23	1.1e-25	3.3e-06	8.0e-06	1.9e-32
Multi Omics Random Forest	2.4e-04	3.5e-39	7.4e-13	5.2e-22	1.0e+00	2.8e-48	5.2e-22	5.2e-22	2.8e-48	2.3e-45	6.8e-01	1.3e-01	2.1e-33	7.7e-11	7.7e-55
Naive Cell Line Mean Predictor	5.5e-41	8.0e-06	5.9e-32	4.4e-23	2.8e-48	1.0e+00	4.4e-23	4.4e-23	1.0e+00	1.3e-01	6.6e-49	8.5e-51	2.4e-11	6.7e-34	1.1e-04
Naive Drug Mean Predictor	7.4e-13	7.7e-12	2.4e-04	1.0e+00	5.2e-22	4.4e-23	1.0e+00	1.0e+00	4.4e-23	2.7e-19	4.4e-23	1.1e-25	3.3e-06	8.0e-06	1.9e-32
Naive Mean Effects Predictor	7.4e-13	7.7e-12	2.4e-04	1.0e+00	5.2e-22	4.4e-23	1.0e+00	1.0e+00	4.4e-23	2.7e-19	4.4e-23	1.1e-25	3.3e-06	8.0e-06	1.9e-32
Naive Predictor	5.5e-41	8.0e-06	5.9e-32	4.4e-23	2.8e-48	1.0e+00	4.4e-23	4.4e-23	1.0e+00	1.3e-01	6.6e-49	8.5e-51	2.4e-11	6.7e-34	1.1e-04
Naive Tissue Mean Predictor	9.1e-38	2.3e-03	2.5e-28	2.7e-19	2.3e-45	1.3e-01	2.7e-19	2.7e-19	1.3e-01	1.0e+00	3.4e-46	2.8e-48	6.1e-08	2.0e-30	1.7e-07
Proteomics Random Forest	4.8e-05	4.2e-40	6.8e-14	4.4e-23	6.8e-01	6.6e-49	4.4e-23	4.4e-23	6.6e-49	3.4e-46	1.0e+00	2.8e-01	2.3e-34	7.7e-12	2.1e-55
Random Forest	4.8e-07	2.6e-42	1.4e-16	1.1e-25	1.3e-01	8.5e-51	1.1e-25	1.1e-25	8.5e-51	2.8e-48	2.8e-01	1.0e+00	8.1e-37	2.0e-14	6.1e-57
SRMF	1.3e-24	8.9e-03	2.0e-14	3.3e-06	2.1e-33	2.4e-11	3.3e-06	3.3e-06	2.4e-11	6.1e-08	2.3e-34	8.1e-37	1.0e+00	1.4e-16	6.3e-21
Simple Neural Network	1.1e-03	4.4e-23	3.9e-01	8.0e-06	7.7e-11	6.7e-34	8.0e-06	8.0e-06	6.7e-34	2.0e-30	7.7e-12	2.0e-14	1.4e-16	1.0e+00	2.6e-42
SuperFELTR	1.5e-48	2.0e-14	1.5e-40	1.9e-32	7.7e-55	1.1e-04	1.9e-32	1.9e-32	1.1e-04	1.7e-07	2.1e-55	6.1e-57	6.3e-21	2.6e-42	1.0e+00
							Test statistic: t-statistic, df=126								
DIPK	0.0	15.702	4.262	8.076	3.813	20.413	8.076	8.076	20.413	18.843	4.262	5.384	13.011	3.365	24.451
Elastic Net	15.702	0.0	11.44	7.627	19.516	4.711	7.627	7.627	4.711	3.14	19.964	21.086	2.692	12.338	8.748
Gradient Boosting	4.262	11.44	0.0	3.813	8.076	16.151	3.813	3.813	16.151	14.581	8.524	9.646	8.748	0.897	20.189
Multi Omics Neural Network	8.076	7.627	3.813	0.0	11.889	12.338	0.0	0.0	12.338	10.767	12.338	13.459	4.935	4.711	16.375
Multi Omics Random Forest	3.813	19.516	8.076	11.889	0.0	24.227	11.889	11.889	24.227	22.656	0.449	1.57	16.824	7.178	28.264
Naive Cell Line Mean Predictor	20.413	4.711	16.151	12.338	24.227	0.0	12.338	12.338	0.0	1.57	24.675	25.797	7.403	17.048	4.038
Naive Drug Mean Predictor	8.076	7.627	3.813	0.0	11.889	12.338	0.0	0.0	12.338	10.767	12.338	13.459	4.935	4.711	16.375
Naive Mean Effects Predictor	8.076	7.627	3.813	0.0	11.889	12.338	0.0	0.0	12.338	10.767	12.338	13.459	4.935	4.711	16.375
Naive Predictor	20.413	4.711	16.151	12.338	24.227	0.0	12.338	12.338	0.0	1.57	24.675	25.797	7.403	17.048	4.038
Naive Tissue Mean Predictor	18.843	3.14	14.581	10.767	22.656	1.57	10.767	10.767	1.57	0.0	23.105	24.227	5.832	15.478	5.608
Proteomics RandomForest	4.262	19.964	8.524	12.338	0.449	24.675	12.338	12.338	24.675	23.105	0.0	1.122	17.273	7.627	28.713
Random Forest	5.384	21.086	9.646	13.459	1.57	25.797	13.459	13.459	25.797	24.227	1.122	0.0	18.394	8.748	29.835
SRMF	13.011	2.692	8.748	4.935	16.824	7.403	4.935	4.935	7.403	5.832	17.273	18.394	0.0	9.646	11.44
Simple Neural Network	3.365	12.338	0.897	4.711	7.178	17.048	4.711	4.711	17.048	15.478	7.627	8.748	9.646	0.0	21.086
SuperFELTR	24.451	8.748	20.189	16.375	28.264	4.038	16.375	16.375	4.038	5.608	28.713	29.835	11.44	21.086	0.0

Table S15: **Statistical test details for the LCO pairwise post-hoc Conover tests.** For all tests, the degrees of freedom are calculated as $nk - k - n + 1 = 126$, where n =number of cross-validation folds, i.e., 10, and k =number of models, i.e., 15. All p-values are multiple testing-adjusted with Benjamini-Hochberg correction. They are derived from the t-statistics given. The post-hoc Conover test is always two-sided.

	DIPK	ENet	GB	MO-NN	MO-RF	CL Mean	Drug Mean	Mean Eff.	Naive Pred	Tissue Mean	Prot. RF	RF	SRMF	Simple-NN
Benjamini-Hochberg adjusted p-values														
DIPK	1.0e+00	4.8e-15	1.1e-01	6.5e-05	6.1e-02	3.5e-19	1.8e-01	1.8e-01	3.5e-19	3.5e-19	1.3e-03	3.8e-05	9.5e-14	8.4e-04
Elastic Net	4.8e-15	1.0e+00	2.9e-11	4.0e-06	1.9e-19	7.9e-02	7.7e-12	7.7e-12	7.9e-02	7.9e-02	1.0e-22	8.9e-25	6.1e-01	1.9e-07
Gradient Boosting	1.1e-01	2.9e-11	1.0e+00	1.6e-02	5.2e-04	2.3e-15	8.1e-01	8.1e-01	2.3e-15	2.3e-15	2.2e-06	2.7e-08	4.7e-10	7.9e-02
Multi Omics Neural Network	6.5e-05	4.0e-06	1.6e-02	1.0e+00	1.4e-08	9.0e-10	7.4e-03	7.4e-03	9.0e-10	9.0e-10	1.6e-11	9.5e-14	3.8e-05	5.2e-01
Multi Omics Random Forest	6.1e-02	1.9e-19	5.2e-04	1.4e-08	1.0e+00	1.2e-23	1.3e-03	1.3e-03	1.2e-23	1.2e-23	1.8e-01	2.2e-02	3.3e-18	3.5e-07
Naive Cell Line Mean Predictor	3.5e-19	7.9e-02	2.3e-15	9.0e-10	1.2e-23	1.0e+00	5.6e-16	5.6e-16	1.0e+00	1.0e+00	1.2e-26	1.4e-28	2.2e-02	2.9e-11
Naive Drug Mean Predictor	1.8e-01	7.7e-12	8.1e-01	7.4e-03	1.3e-03	5.6e-16	1.0e+00	1.0e+00	5.6e-16	5.6e-16	7.0e-06	9.9e-08	1.2e-10	4.4e-02
Naive Mean Effects Predictor	1.8e-01	7.7e-12	8.1e-01	7.4e-03	1.3e-03	5.6e-16	1.0e+00	1.0e+00	5.6e-16	5.6e-16	7.0e-06	9.9e-08	1.2e-10	4.4e-02
Naive Predictor	3.5e-19	7.9e-02	2.3e-15	9.0e-10	1.2e-23	1.0e+00	5.6e-16	5.6e-16	1.0e+00	1.0e+00	1.2e-26	1.4e-28	2.2e-02	2.9e-11
Naive Tissue Mean Predictor	3.5e-19	7.9e-02	2.3e-15	9.0e-10	1.2e-23	1.0e+00	5.6e-16	5.6e-16	1.0e+00	1.0e+00	1.2e-26	1.4e-28	2.2e-02	2.9e-11
Proteomics Random Forest	1.3e-03	1.0e-22	2.2e-06	1.6e-11	1.8e-01	1.2e-26	7.0e-06	7.0e-06	1.2e-26	1.2e-26	1.0e+00	3.5e-01	2.1e-21	4.7e-10
Random Forest	3.8e-05	8.9e-25	2.7e-08	9.5e-14	2.2e-02	1.4e-28	9.9e-08	9.9e-08	1.4e-28	1.4e-28	3.5e-01	1.0e+00	1.2e-23	3.9e-12
SRMF	9.5e-14	6.1e-01	4.7e-10	3.8e-05	3.3e-18	2.2e-02	1.2e-10	1.2e-10	2.2e-02	2.2e-02	2.1e-21	1.2e-23	1.0e+00	2.2e-06
Simple Neural Network	8.4e-04	1.9e-07	7.9e-02	5.2e-01	3.5e-07	2.9e-11	4.4e-02	4.4e-02	2.9e-11	2.9e-11	4.7e-10	3.9e-12	2.2e-06	1.0e+00
Test statistic: t-statistic, df=117														
DIPK	0.0	9.222	1.703	4.256	1.986	11.067	1.419	1.419	11.067	11.067	3.405	4.398	8.655	3.547
Elastic Net	9.222	0.0	7.52	4.966	11.209	1.844	7.804	7.804	1.844	1.844	12.628	13.621	0.568	5.675
Gradient Boosting	1.703	7.52	0.0	2.554	3.689	9.364	0.284	0.284	9.364	9.364	5.108	6.101	6.952	1.844
Multi Omics Neural Network	4.256	4.966	2.554	0.0	6.243	6.81	2.838	2.838	6.81	6.81	7.662	8.655	4.398	0.709
Multi Omics Random Forest	1.986	11.209	3.689	6.243	0.0	13.053	3.405	3.405	13.053	13.053	1.419	2.412	10.641	5.533
Naive Cell Line Mean Predictor	11.067	1.844	9.364	6.81	13.053	0.0	9.648	9.648	0.0	0.0	14.472	15.465	2.412	7.52
Naive Drug Mean Predictor	1.419	7.804	0.284	2.838	3.405	9.648	0.0	0.0	9.648	9.648	4.824	5.817	7.236	2.128
Naive Mean Effects Predictor	1.419	7.804	0.284	2.838	3.405	9.648	0.0	0.0	9.648	9.648	4.824	5.817	7.236	2.128
Naive Predictor	11.067	1.844	9.364	6.81	13.053	0.0	9.648	9.648	0.0	0.0	14.472	15.465	2.412	7.52
Naive Tissue Mean Predictor	11.067	1.844	9.364	6.81	13.053	0.0	9.648	9.648	0.0	0.0	14.472	15.465	2.412	7.52
Proteomics Random Forest	3.405	12.628	5.108	7.662	1.419	14.472	4.824	4.824	14.472	14.472	0.0	0.993	12.06	6.952
Random Forest	4.398	13.621	6.101	8.655	2.412	15.465	5.817	5.817	15.465	15.465	0.993	0.0	13.053	7.945
SRMF	8.655	0.568	6.952	4.398	10.641	2.412	7.236	7.236	2.412	2.412	12.06	13.053	0.0	5.108
Simple Neural Network	3.547	5.675	1.844	0.709	5.533	7.52	2.128	2.128	7.52	7.52	6.952	7.945	5.108	0.0

Table S16: **Statistical test details for the LTO pairwise post-hoc Conover tests.** For all tests, the degrees of freedom are calculated as $nk - k - n + 1 = 126$, where n =number of cross-validation folds, i.e., 10, and k =number of models, i.e., 14. All p-values are multiple testing-adjusted with Benjamini-Hochberg correction. They are derived from the t-statistics given. The post-hoc Conover test is always two-sided.

	DIPK	ENet	GB	MO-NN	MO-RF	Mean Eff.	Naive Pred	Tissue Mean	Prot. RF	RF	Simple-NN
					Benjamini-Hochberg adjusted p-values						
DIPK	1.0	0.857	0.713	0.713	0.762	0.857	0.258	0.577	0.762	0.285	0.98
Elastic Net	0.857	1.0	0.577	0.577	0.857	0.762	0.299	0.713	0.857	0.372	0.857
Gradient Boosting	0.713	0.577	1.0	1.0	0.441	0.762	0.1	0.299	0.441	0.1	0.738
Multi Omics Neural Network	0.713	0.577	1.0	1.0	0.441	0.762	0.1	0.299	0.441	0.1	0.738
Multi Omics Random Forest	0.762	0.857	0.441	0.441	1.0	0.711	0.43	0.762	1.0	0.441	0.762
Naive Mean Effects Predictor	0.857	0.762	0.762	0.762	0.711	1.0	0.207	0.441	0.711	0.245	0.899
Naive Predictor	0.258	0.299	0.1	0.1	0.43	0.207	1.0	0.577	0.43	0.94	0.249
Naive Tissue Mean Predictor	0.577	0.713	0.299	0.299	0.762	0.441	0.577	1.0	0.762	0.688	0.577
Proteomics Random Forest	0.762	0.857	0.441	0.441	1.0	0.711	0.43	0.762	1.0	0.441	0.762
Random Forest	0.285	0.372	0.1	0.1	0.441	0.245	0.94	0.688	0.441	1.0	0.27
Simple Neural Network	0.98	0.857	0.738	0.738	0.762	0.899	0.249	0.577	0.762	0.27	1.0
					Test statistic: t-statistic, df=90						
DIPK	0.0	0.282	0.774	0.774	0.563	0.282	2.111	1.056	0.563	1.971	0.07
Elastic Net	0.282	0.0	1.056	1.056	0.282	0.563	1.83	0.774	0.282	1.689	0.352
Gradient Boosting	0.774	1.056	0.0	0.0	1.337	0.493	2.886	1.83	1.337	2.745	0.704
Multi Omics Neural Network	0.774	1.056	0.0	0.0	1.337	0.493	2.886	1.83	1.337	2.745	0.704
Multi Omics Random Forest	0.563	0.282	1.337	1.337	0.0	0.845	1.548	0.493	0.0	1.408	0.633
Naive Mean Effects Predictor	0.282	0.563	0.493	0.493	0.845	0.0	2.393	1.337	0.845	2.252	0.211
Naive Predictor	2.111	1.83	2.886	2.886	1.548	2.393	0.0	1.056	1.548	0.141	2.182
Naive Tissue Mean Predictor	1.056	0.774	1.83	1.83	0.493	1.337	1.056	0.0	0.493	0.915	1.126
Proteomics Random Forest	0.563	0.282	1.337	1.337	0.0	0.845	1.548	0.493	0.0	1.408	0.633
Random Forest	1.971	1.689	2.745	2.745	1.408	2.252	0.141	0.915	1.408	0.0	2.041
Simple Neural Network	0.07	0.352	0.704	0.704	0.633	0.211	2.182	1.126	0.633	2.041	0.0

Table S17: **Statistical test details for the LDO pairwise post-hoc Conover tests.** For all tests, the degrees of freedom are calculated as $nk - k - n + 1 = 90$, where n =number of cross-validation folds, i.e., 10, and k =number of models, i.e., 11. All p-values are multiple testing-adjusted with Benjamini-Hochberg correction. They are derived from the t-statistics given. The post-hoc Conover test is always two-sided.

References

[1] Fangfang Xia, Jonathan Allen, Prasanna Balaprakash, Thomas Brettin, Cristina Garcia-Cardona, Austin Clyde, Judith Cohn, James Doroshow, Xiaotian Duan, Veronika Dubinkina, Yvonne Evrard, Ya Ju Fan, Jason Gans, Stewart He, Pinyi Lu, Sergei Maslov, Alexander Partin, Maulik Shukla, Eric

Stahlberg, Justin M Wozniak, Hyunseung Yoo, George Zaki, Yitan Zhu, and Rick Stevens. A cross-study analysis of drug response prediction in cancer cell lines. *Briefings in Bioinformatics*, 23(1):bbab356, January 2022.

[2] Alexander Partin, Thomas S. Brettin, Yitan Zhu, Oleksandr Narykov, Austin Clyde, Jamie Overbeek, and Rick L. Stevens. Deep learning methods for drug response prediction in cancer: Predominant and emerging trends. *Frontiers in Medicine*, 10, February 2023. Publisher: Frontiers.

[3] Edward H Simpson. The interpretation of interaction in contingency tables. *Journal of the Royal Statistical Society: Series B (Methodological)*, 13(2):238–241, 1951.

[4] Seyone Chithrananda, Gabriel Grand, and Bharath Ramsundar. Chemberta: large-scale self-supervised pretraining for molecular property prediction. *arXiv preprint arXiv:2010.09885*, 2020.

[5] Sunghwan Kim, Jie Chen, Tiejun Cheng, Asta Gindulyte, Jia He, Siqian He, Qingliang Li, Benjamin A Shoemaker, Paul A Thiessen, Bo Yu, et al. Pubchem 2025 update. *Nucleic Acids Research*, 53(D1):D1516–D1525, 2025.

[6] William G. Herbert, Nicholas Chia, Paul A. Jensen, and Marina RS Walther-Antonio. Monotherapy cancer drug-blind response prediction is limited to intraclass generalization. *bioRxiv*, 2025.

[7] Pavel G Polishchuk, Timur I Madzhidov, and Alexandre Varnek. Estimation of the size of drug-like chemical space based on gdb-17 data. *Journal of computer-aided molecular design*, 27(8):675–679, 2013.

[8] John J Irwin, Teague Sterling, Michael M Mysinger, Erin S Bolstad, and Ryan G Coleman. Zinc: a free tool to discover chemistry for biology. *Journal of chemical information and modeling*, 52(7):1757–1768, 2012.

[9] Karim ElSawy, Chandra S Verma, David P Lane, and Leo Caves. On the origin of the stereoselective affinity of nutlin-3 geometrical isomers for the mdm2 protein. *Cell Cycle*, 12(24):3727–3735, 2013.

[10] Oleksandr Narykov, Yitan Zhu, Thomas Brettin, Yvonne A Evrard, Alexander Partin, Fangfang Xia, Maulik Shukla, Priyanka Vasanthakumari, James H Doroshow, and Rick L Stevens. Data imbalance in drug response prediction: multi-objective optimization approach in deep learning setting. *Briefings in Bioinformatics*, 26(2), 2025.

[11] Qianrong Guo, Saiveth Hernandez-Hernandez, and Pedro J Ballester. Scaffold splits overestimate virtual screening performance. In *International Conference on Artificial Neural Networks*, pages 58–72. Springer, 2024.

[12] Mahmoud Ghandi, Franklin W Huang, Judit Jané-Valbuena, Gregory V Kryukov, Christopher C Lo, E Robert McDonald III, Jordi Barretina, Ellen T Gelfand, Craig M Bielski, Haoxin Li, et al. Next-generation characterization of the cancer cell line encyclopedia. *Nature*, 569(7757):503–508, 2019.

[13] Emanuel Gonçalves, Rebecca C Poulos, Zhaoxiang Cai, Syd Barthorpe, Srikanth S Manda, Natasha Lucas, Alexandra Beck, Daniel Bucio-Noble, Michael Dausmann, Caitlin Hall, et al. Pan-cancer proteomic map of 949 human cell lines. *Cancer cell*, 40(8):835–849, 2022.

[14] Daniel Bottomly, Nicholas Long, Andrew R. Schultz, Sean E. Kurtz, Christine E. Tognon, Kelsie Johnson, and Jeffrey W. Tyner. Beataml2 dataset: Integrative analysis of drug response and clinical outcome in acute myeloid leukemia. `https://biodev.github.io/BeatAML2/`, 2022. GitHub Pages.

[15] Alejandra Bruna, Oscar M Rueda, Wendy Greenwood, Ankita Sati Batra, Maurizio Callari, Rajbir Nath Batra, Katherine Pogrebniak, Jose Sandoval, John W Cassidy, Ana Tufegdzic-Vidakovic, et al. A biobank of breast cancer explants with preserved intra-tumor heterogeneity to screen anticancer compounds. *Cell*, 167(1):260–274, 2016.

[16] Akshat Singhal, Xiaoyu Zhao, Patrick Wall, Emily So, Guido Calderini, Alexander Partin, Natasha Koussa, Priyanka Vasanthakumari, Oleksandr Narykov, Yitan Zhu, et al. The hallmarks of predictive oncology. *Cancer Discovery*, 15(2):271–285, 2025.
