# Supplementary material for: Critical evaluation of drug response prediction models with DrEval
Source: Nat Commun. 2026 May 12;17:4238. doi: 10.1038/s41467-026-72903-w (PMC13168506; doi:10.1038/s41467-026-72903-w)
Supplement: Supplementary file 2 — Description of Additional Supplementary Files [file 41467_2026_72903_MOESM2_ESM.pdf]

Description of Additional Supplementary Files

- Supplementary Data 1: A detailed comparison of DrEval to prior benchmarking efforts.
- Supplementary Data 2: An overview of all models currently implemented in DrEval.
