# Supplementary material for: Critical evaluation of drug response prediction models with DrEval
Source: Nat Commun. 2026 May 12;17:4238. doi: 10.1038/s41467-026-72903-w (PMC13168506; doi:10.1038/s41467-026-72903-w)
Supplement: Supplementary file 4 — Supplementary Data 2 [file 41467_2026_72903_MOESM4_ESM.pdf]

Supplementary Data 2

Overview of the models implemented in DrEval. Baseline methods were implemented by us, while published methods were published before, but re-implemented by us for our framework. Global models have one set of hyperparameters and can generalize to unseen drugs, while single-drug models tune one model per drug and cannot generalize to unseen drugs.

Model	Baseline / Published	Global / Single-Drug	Description
NaivePredictor	Baseline Method	Global Model	Predicts the mean response of all drugs in the training set.
NaiveCellLine-MeanPredictor	Baseline Method	Global Model	Predicts the mean response of a cell line in the training set.
NaiveTissue-MeanPredictor	Baseline Method	Global Model	Predicts the mean response of a tissue of origin in the training set.
NaiveDrug-MeanPredictor	Baseline Method	Global Model	Predicts the mean response of a drug in the training set.
NaiveMean-EffectPredictor	Baseline Method	Global Model	Predicts mean of dataset + mean of cell line + mean of drug
ElasticNet	Baseline Method	Global Model	Fits an Sklearn Elastic Net on gene expression data and drug fingerprints.
Single-Drug Elastic Net	Baseline Method	Single-Drug Model	Fits an Sklearn Elastic Net on gene expression data for each drug separately.
Single-Drug Proteomics Elastic Net	Baseline Method	Single-Drug Model	Fits an Sklearn Elastic Net on proteomics data for each drug separately.
Gradient-Boosting	Baseline Method	Global Model	Fits an Sklearn Histogram-based Gradient Boosting Regression Tree on gene expression data and drug fingerprints.
RandomForest	Baseline Method	Global Model	Fits an Sklearn Random Forest Regressor on gene expression data and drug fingerprints.
Proteomics Random Forest	Baseline Method	Global Model	Fits an Sklearn Random Forest Regressor on protein expression data and drug fingerprints.
MultiOmics-RandomForest	Baseline Method	Global Model	Fits an Sklearn Random Forest Regressor on gene expression, methylation, mutation, copy number variation data, and drug fingerprints (concatenated matrix). The dimensionality of the methylation data is reduced with a PCA to the first 100 components before it is fed to the model.
SingleDrug-RandomForest	Baseline Method	Single-Drug Model	Fits an Sklearn Random Forest Regressor on gene expression data for each drug separately.
SVR	Baseline Method	Global Model	Fits an Sklearn Support Vector Regressor on gene expression data and drug fingerprints.
Simple-NeuralNetwork	Baseline Method	Global Model	Fits a feedforward neural network on gene expression and drug fingerprints with 3 layers + dropout

MultiOmics-NeuralNetwork	Baseline Method	Global Model	Fits a feedforward neural network on gene expression, methylation, mutation, copy number variation data, and drug fingerprints, 3 layers + dropout. The dimensionality of the methylation data is PCA-reduced before it is fed to the model.
ChemBERTa-NeuralNetwork	Baseline Method	Global Model	Fits a feedforward neural network on gene expression data and ChemBERTa drug embeddings (large pre-trained transformer representations of SMILES strings). The network architecture and training procedure are identical to the SimpleNeuralNetwork.
DrugGNN	Baseline Method	Global Model	Graph neural network for drug response prediction by encoding each drug as a molecular graph (Graph-convolutional layers with global pooling) and each cell line as a vector of gene expression features (feed-forward encoder).
SRMF	Published Model	Global Model	Similarity Regularization Matrix Factorization model by Wang et al. on gene expression data and drug fingerprints. Similarities to all other drugs/cell lines represent each drug and cell line and are mapped into a shared latent low-dimensional space from which responses are predicted.
MOLIR	Published Model	Single-Drug Model	Regression extension of MOLI: multi-omics late integration deep neural network by Sharifi-Noghabi et al. Takes mutation, copy number variation, and gene expression data as input. MOLI reduces the dimensionality of each omics type with a hidden layer, concatenates them into one representation, and optimizes this representation via a combined cost function consisting of a triplet loss and a binary cross-entropy loss. We implemented a regression adaption with MSE loss and an adapted triplet loss for regression.
SuperFELTR	Published Model	Single-Drug Model	Regression extension of SuperFELT: supervised feature extraction learning using triplet loss for drug response by Park et al. Very similar to MOLI(R). In MOLI(R), encoders and the classifier were trained jointly. SuperFELT(R) trains them independently.
DIPK	Published Model	Global Model	Deep Neural Network Integrating Prior Knowledge from Li et al. Uses gene interaction relationships (encoded by a graph auto-encoder), gene expression profiles (encoded by a denoising auto-encoder), and molecular topologies (encoded by MolGNet). Those features are integrated using multi-head attention layers.
