# Supplementary material for: Critical evaluation of drug response prediction models with DrEval
Source: Nat Commun. 2026 May 12;17:4238. doi: 10.1038/s41467-026-72903-w (PMC13168506; doi:10.1038/s41467-026-72903-w)
Supplement: Supplementary file 5 — Reporting Summary [file 41467_2026_72903_MOESM5_ESM.pdf]

nature portfolio

Corresponding author(s): Dr. Markus List

Last updated by author(s): Jan 20, 2026

Reporting Summary

Nature Portfolio wishes to improve the reproducibility of the work that we publish. This form provides structure for consistency and transparency in reporting. For further information on Nature Portfolio policies, see our Editorial Policies and the Editorial Policy Checklist.

Statistics

For all statistical analyses, confirm that the following items are present in the figure legend, table legend, main text, or Methods section.

n/a	Confirmed	
☐	☒	The exact sample size (*n*) for each experimental group/condition, given as a discrete number and unit of measurement
☐	☒	A statement on whether measurements were taken from distinct samples or whether the same sample was measured repeatedly
☐	☒	The statistical test(s) used AND whether they are one- or two-sided<br>*Only common tests should be described solely by name; describe more complex techniques in the Methods section.*
☐	☒	A description of all covariates tested
☐	☒	A description of any assumptions or corrections, such as tests of normality and adjustment for multiple comparisons
☐	☒	A full description of the statistical parameters including central tendency (e.g. means) or other basic estimates (e.g. regression coefficient) AND variation (e.g. standard deviation) or associated estimates of uncertainty (e.g. confidence intervals)
☐	☒	For null hypothesis testing, the test statistic (e.g. *F*, *t*, *r*) with confidence intervals, effect sizes, degrees of freedom and *P* value noted<br>*Give P values as exact values whenever suitable.*
☒	☐	For Bayesian analysis, information on the choice of priors and Markov chain Monte Carlo settings
☒	☐	For hierarchical and complex designs, identification of the appropriate level for tests and full reporting of outcomes
☐	☒	Estimates of effect sizes (e.g. Cohen's *d*, Pearson's *r*), indicating how they were calculated

Our web collection on statistics for biologists contains articles on many of the points above.

Software and code

Policy information about availability of computer code

Data collection	All details regarding the data collection are documented with all necessary code at https://github.com/daisybio/preprocess_drp_data (v.1.0.0)
Data analysis	All details regarding the data analysis are documented with all necessary code at https://github.com/daisybio/drevalpy (v.1.4.1, DOI: 10.5281/zenodo.18302237) and https://github.com/nf-core/drugresponseeval (v1.2.0, DOI: 10.5281/zenodo.14779984).

For manuscripts utilizing custom algorithms or software that are central to the research but not yet described in published literature, software must be made available to editors and reviewers. We strongly encourage code deposition in a community repository (e.g. GitHub). See the Nature Portfolio guidelines for submitting code & software for further information.

Data

Policy information about availability of data

All manuscripts must include a data availability statement. This statement should provide the following information, where applicable:

- Accession codes, unique identifiers, or web links for publicly available datasets
- A description of any restrictions on data availability
- For clinical datasets or third party data, please ensure that the statement adheres to our policy

The drug response, cell line, and drug data used in this study are available in the Zenodo database under accession code 12633909 (DOI: 10.5281/zenodo.12633909). Curve fits for the cell line datasets can be explored at ProteomicsDB: https://www.proteomicsdb.org/analytics/cellSensitivity. The data underlying all Figures and Tables are provided in the Source Data file.

Research involving human participants, their data, or biological material

Policy information about studies with human participants or human data. See also policy information about sex, gender (identity/presentation), and sexual orientation and race, ethnicity and racism.

Reporting on sex and gender	No information regarding sex and gender has been collected and we did not conduct any sex- or gender-stratified drug response prediction experiments. We expect this to become relevant once the models considerably outperform naive predictors.
Reporting on race, ethnicity, or other socially relevant groupings	No information regarding race, ethnicity, or other socially relevant groupings has been collected and we did not conduct any drug response prediction experiments stratified accordingly. We expect this to become relevant once the models considerably outperform naive predictors.
Population characteristics	n.a.
Recruitment	n.a.
Ethics oversight	n.a.

Note that full information on the approval of the study protocol must also be provided in the manuscript.

Field-specific reporting

Please select the one below that is the best fit for your research. If you are not sure, read the appropriate sections before making your selection.

☒ Life sciences ☐ Behavioural & social sciences ☐ Ecological, evolutionary & environmental sciences

For a reference copy of the document with all sections, see nature.com/documents/nr-reporting-summary-flat.pdf

Life sciences study design

All studies must disclose on these points even when the disclosure is negative.

Sample size	No statistical method was used to predetermine sample size. We used all available drug–cell line pairs from the CTRPv2 dataset (approximately 400,000 response curves across 886 cell lines and 545 compounds) as the primary training and evaluation dataset, which is the largest publicly available drug sensitivity screen. We note that the effective sample size for generalization is limited by the number of unique biological and chemical entities rather than the number of drug–cell line pairs, and we discuss this as the pseudoreplication problem in our study. For the cross-validation, a sample size of 10 was set to balance statistical power and computational cost.
Data exclusions	Dataset selection:<br>Seven publicly available drug response datasets were included in this study: CCLE, CTRPv1, CTRPv2, GDSC1, GDSC2, BeatAML2, and PDX_Bruna. Other existing datasets were not included in the initial version of DrEval for specific reasons. The NCI-60 dataset was excluded due to its small panel size of only 60 cell lines. The PRISM dataset was not included because it uses a different assay methodology based on multiplexed barcoding, which is incompatible with our CurveCurator-based standardization of response metrics. The gCSI dataset was excluded because it contains a comparatively small drug panel of approximately 44 compounds. This dataset selection criterion was not pre-established but mainly reflects time constraints. We plan to extend the number of included datasets in future updates.<br><br>Drug response data quality filtering:<br>EC50 values that fell outside the measured dosage range for a given drug and IC50 values that deviated by more than one order of magnitude from the measured range were considered invalid and excluded from analysis. This criterion was pre-established.<br><br>Feature data filtering:<br>Due to limited overlap between RNA sequencing and RRBS methylation data in GDSC1 and GDSC2, these datasets were paired instead with microarray-based gene expression and BeadChip methylation data. Cell lines lacking matching omics data were necessarily excluded from all analyses. This was a pre-established practical constraint. Mutation data were filtered to include only non-silent coding mutations, ensuring that only potentially functional variants were considered as features. Proteomics data were filtered based on a global Q value threshold of 0.01 and restricted to proteotypic peptides. This pre-established quality cutoff ensures reliable estimation of protein abundance.<br><br>Cell line and drug identifier mapping:<br>No exclusions.
Replication	All experiments were evaluated using 10-fold cross-validation, ensuring that each data point appeared in the test set exactly once. Cross-study generalization was assessed by training on CTRPv2 and predicting on independent datasets (CTRPv1, CCLE, GDSC1, GDSC2, BeatAML2, PDX_Bruna). All results, including per-fold metrics and standard errors, are reported and fully reproducible via a single command using the DrEval pipeline (drevalpy Python package or nf-core/drugresponseeval Nextflow pipeline).
Randomization	We perform nested cross-validation, i.e., we split the data into 10 cross-validation folds and then, we use 10% of each training fold as validation data. When the model requires one, we split an early stopping set from the validation set that is usually 1/4th of the validation data, if there is enough data (if not, then 1/3rd or half). The randomization depends on the setting. For LPO, we split the data randomly. For LCO, LTO, and LDO, we do a random grouped split with group=cell line, tissue, or drug ID.

Blinding

Blinding is not applicable to this computational study as there were no experimental groups. Assignment to training, validation, early stopping, and test dataset was performed randomly, as described above, by sklearn.

Reporting for specific materials, systems and methods

We require information from authors about some types of materials, experimental systems and methods used in many studies. Here, indicate whether each material, system or method listed is relevant to your study. If you are not sure if a list item applies to your research, read the appropriate section before selecting a response.

Materials & experimental systems

n/a	Involved in the study	
☒	☐	Antibodies
☒	☐	Eukaryotic cell lines
☒	☐	Palaeontology and archaeology
☒	☐	Animals and other organisms
☒	☐	Clinical data
☒	☐	Dual use research of concern
☒	☐	Plants

Methods

n/a	Involved in the study	
☒	☐	ChIP-seq
☒	☐	Flow cytometry
☒	☐	MRI-based neuroimaging

Plants

Seed stocks

Report on the source of all seed stocks or other plant material used. If applicable, state the seed stock centre and catalogue number. If plant specimens were collected from the field, describe the collection location, date and sampling procedures.

Novel plant genotypes

Describe the methods by which all novel plant genotypes were produced. This includes those generated by transgenic approaches, gene editing, chemical/radiation-based mutagenesis and hybridization. For transgenic lines, describe the transformation method, the number of independent lines analyzed and the generation upon which experiments were performed. For gene-edited lines, describe the editor used, the endogenous sequence targeted for editing, the targeting guide RNA sequence (if applicable) and how the editor was applied.

Authentication

Describe any authentication procedures for each seed stock used or novel genotype generated. Describe any experiments used to assess the effect of a mutation and, where applicable, how potential secondary effects (e.g. second site T-DNA insertions, mosiacism, off-target gene editing) were examined.
